# RSM integrated GWO, Driving Training, and Election-Based Algorithms for optimising ethylic biodiesel from ternary oil of neem, animal fat, and jatropha

**DOI:** 10.1038/s41598-024-72109-4

**Published:** 2024-09-12

**Authors:** Olusegun D. Samuel, G. C. Manjunath Patel, Likewin Thomas, Davannendran Chandran, Prabhu Paramasivam, Christopher C. Enweremadu

**Affiliations:** 1 Department of Mechanical Engineering, Federal University of Petroleum Resources, P.M.B 1221, Effurun, Delta State Nigeria; 2 Department of Mechanical Engineering, PES Institute of Technology and Management, Visvesvaraya Technological University, Shivamogga, 577204 Karnataka India; 3 Department of Artificial Intelligence and Machine Learning, PES Institute of Technology and Management, Visvesvaraya Technological University, Shivamogga, 577204 Karnataka India; 4 Department of Mechanical Engineering, Universiti Teknologi PETRONAS, 32610 Seri Iskandar, Perak, Malaysia; 5 Department of Research and Innovation, Saveetha School of Engineering, SIMATS, Chennai, Tamil Nadu 602105 India; 6 Department of Mechanical Engineering, College of Engineering and Technology, Mattu University, Mettu, Ethiopia; 7 Department of Mechanical, Bioresources and Biomedical Engineering, Science Campus, University of South Africa, Private Bag X6, Florida, 1709 South Africa

**Keywords:** DTBO, EBOA, GWO, Neem, Fat, Jatropha seed oil, RSM, Biodiesel, Bioenergy, Environmental impact

## Abstract

The worldwide exploration of the ethanolysis protocol (EP) has decreased despite the multifaceted benefits of ethanol, such as lower toxicity, higher oxygen content, higher renewability, and fewer emission tail compared to methanol, and the enhanced fuel properties with improved engine characteristics of multiple-oily feedstocks (MOFs) compared to single-oily feedstocks. The study first proposed a strategy for the optimisation of ethylic biodiesel synthesis from MOFs: neem, animal fat, and jatropha oil (NFJO) on a batch reactor. The project's goals were to ensure environmental benignity and encourage the use of totally biobased products. This was made possible by the introduction of novel population based algorithms such as Driving Training-Based Optimization (DTBO) and Election-Based Optimization (EBOA), which were compared with the widely used Grey Wolf Optimizer (GWO) combined with Response Surface Methodology (RSM). The yield of NFJO ethyl ester (NFJOEE) was predicted using the RSM technique, and the ideal transesterification conditions were determined using the DTBO, EBOA, and GWO algorithms. Reaction time showed a strong linear relationship with ethylic biodiesel yield, while ethanol-to-NFJO molar ratio, catalyst dosage, and reaction temperature showed nonlinear effects. Reaction time was the most significant contributor to NFJOEE yield.The important fundamental characteristics of the fuel categories were investigated using the ASTM test procedures. The maximum NFJOEE yield (86.3%) was obtained at an ethanol/NFJO molar ratio of 5.99, KOH content of 0.915 wt.%, ethylic duration of 67.43 min, and reaction temperature of 61.55 °C. EBOA outperforms DTBO and GWO regarding iteration and computation time, converging towards a global fitness value equal to 7 for 4 s, 20 for 5 s and 985 for 34 s. The key fuel properties conformed to the standards outlined by ASTMD6751 and EN 14,214 specifications. The NFJOEE fuel processing cost is 0.9328 USD, and is comparatively lesser than that of conventional diesel. The new postulated population based algorithm models can be a prospective approach for enhancing biodiesel production from numerous MOFs and ensuring a balanced ecosystem and fulfilling enviromental benignity when adopted.

## Introduction

The higher global accolade recoded by the ethylic protocol compared to methylic approach can be linked with lower toxicity, higher oxygen content, higher renewability, and fewer emission tails. The energy crisis, along with fossil fuel depletion and global warming has forced the transportation sector to use clean fuels (liquids or gases) that produce zero or low carbon emissions in engines^1^. Further utilisation of binary oil feedstocks (BOFs) and ternary oil feedstocks (TOFs) over single oil feedstocks (SOF) by researchers and biofuel policymakers has been attributed to the need to eradicate fuel versus food competition and enrich fuel-related properties. Furthermore, researchers and policymakers in the biofuel industry need to focus on utilising binary and ternary oil feedstocks to ensure sustainability and increased supply for biodiesel production.

There is a lack of information on TOFs, despite the scarcity of information on BOFs, such as cotton, soybean/castor seeds^2^, castor/karanja^3,4^, beauty leaf/castor seed^5^, soybean/castor seed^6^, and castor seed/waste fish^7^. Currently, biodiesel made from TOFs of animal fat, cotton seed, and rice bran is documented^8^. It is crucial to investigate sustainable biodiesel production from MOFs for the ethylic protocol in light of the impending global commercialization of lower carbon fuel for the biodiesel sector, biofuel vehicle diesel engines, and environmental benefits.

Researchers' interest in ternary based biodiesel production (TBBD) has been piqued by the variety of feedstocks that are readily available globally. One of the most important requirements for a feedstock to be used in TBBD is that it has the ability to be manufactured on a large scale with low production costs. Feedstock supply is influenced by local soil characteristics, geographic location, weather patterns, and agricultural techniques^9^. The utilisation of less expensive multiple feedstocks makes biodiesel manufacturing competitive, cost-effective, and enhances fuel-related properties. Additionally, undesirable by-products have been produced from heterogeneous-based catalysts employed in biodiesel production, leading to an energy crisis and environmental pollution^10^. However, other researchers^11–14^ have attributed the wide application of heterogeneous catalysts to their recycling potential, lower production costs, reusability, and environmentally friendly operation.

Researchers and stakeholders currently favour exploring a combination of second-generation feedstocks (SGFs) to produce enhanced novel green diesel in order to improve the fuel's oxidation stability, density, viscosity, flash point, and other characteristics. Fat, along with neem and jatropha oil, is preferred among SGFs because it has key characteristics similar to diesel fuel. Compared to vegetable oils, animal fats (AFs) from slaughterhouses and poultry farms, such as beef tallow and chicken fat, provide a higher cetane number, contributing to greater combustion heat in biodiesel^15^. AFs have higher levels of saturated fat than VOs, which can lead to undesired oxidation stability during combustion^16^. Moreover, most AFs-based biodiesel has a higher viscosity and remains solid at room temperature^17^. The (m) ethylic reactions in AFs are limited by higher levels of saturated fatty acids (SFAs). High SFAs also result in low-quality byproducts, such as glycerine, and reduce earnings for biodiesel plants^18^. Therefore, in order to scale the biofuel industry and ensure adequate vehicle use, criteria like ease of processing, improved fuel-related characteristics, and abundant availability at low cost must be prioritised. Investigating hybrid-second generation oily feedstocks (HSGOFs), which are commercially feasible, can help address the aforementioned shortcomings related to AFs-dominated feedstock. Given that HSGOFs have been shown to offer superior fuel qualities to traditional diesel, a blend of neem and jatropha oil has shown potential for biodiesel synthesis. Samuel et al.^19^ proposed that hybrid oils could be a viable option for enhancing fuel qualities and engine characteristics by combining the key fuel attributes of each unique oil during the biodiesel production process. The above literature confirms that biodiesel derived from AFs possesses better combustion characteristics (although there is negativity with methylic reaction and low-byproduct quality), and a blend of neem and jatropha offers favorable biodiesel synthesis and fuel characteristics. The combination of oils derived from multiple oily (AFs, neem, and jatropha) feedstocks with ethanolysis protocol has yet to be investigated in the literature.

Neem is a versatile tree that is planted in more than 72 countries worldwide^20^. It is found in both Asia and African countries^21^. In 2023, India topped the charts as the largest producer and exporter with 15,473 shipments of neem oil worldwide. Following India, Sri Lanka secured the second position with 266 shipments, while China was the third-largest exporter, closely behind with 264 shipments^22^. An enormous amount of waste is produced every year by the neem tree's production of neem products. Pollution in the environment needs to be decreased^23^. Produced in millions of metric tons worldwide, neem cake (NC) is a by-product of neem oil, which is made by cold pressing neem kernels^24^. Despite the fact that NC is utilised as animal feed or fertiliser in agriculture and is considered a byproduct with minimal chemical interest, the use of NC is not recommended as they are not economically viable and can be diverted for biofuel production^25^. By utilising soxhlet extraction, more oil may be recovered from NC and used to produce biofuel^26,27^. The increased availability of neem in distinct geographical locations, coupled with the commercial use of its by-products, ensures economically viable feedstocks for biodiesel production. Due to its versatility in many environments, two-year gestation period, high oil yield, and capacity to preserve soil, jatropha makes an ideal feedstock^28^. Additionally, neem and Jatropha waste for the production of low carbon are of profound research curiosity throughout the globe. Shortest ignition delays are guaranteed by hybridizing oils from neem and Jatropha oil (NJO) and biodiesel made from NJO when used in internal combustion (IC) engines. This has been shown to reduce carbon deposits and poor cooking^29^. The literature above confirms that using hybrid NJO biodiesel reduces ignition delay in IC engines, offering a promising low-carbon alternative for biodiesel production.

Not long ago, a trial-and-error experimental approach and a one-variable-at-a-time (OVAT) method have been adopted to optimise biodiesel from NJO. However, due to its inability to correlate response and input variables and its failure to build a reliable forecasting model, the OVAT method has not significantly improved productivity^30^. Several approaches have been suggested to enhance, scale up the production and ensure sustainability. For example, Osman et al.^10^ reported that the application of machine learning, computational chemistry, and data mining can boost yield and optimize the production process. Additionally, a successful circular economy can be achieved by integrating hydrothermal and biochemical routes^10,31^. Therefore, the choice of biodiesel production method for multiple oily feedstocks (AFs, neem, and Jatropha) using the ethanolysis protocol requires significant attention, including appropriate experimental and machine learning techniques, to enhance biodiesel conversion and yield.

### Feedstocks of mixed oils for the production of biodiesel and its transesterification

A technique in which several oily feedstocks are blended together to enhance and complement each individual oil's greatest qualities is referred to as hybrid oils or mixtures of oils. Numerous other physicochemical characteristics, such as kinematic viscosity, acid value, cold flow characteristics, oxidation stability, etc., may also be improved by mixing. In addition to lowering the cost of raw materials, mixing ensures their availability, which lowers manufacturing costs and opens up the possibility of large-scale production. Without the need for additives, the oxidation stability and cold flow properties can be developed by the produced biodiesel made from the blend of oil feedstocks. The idea of blending high- and low-viscosity oils results in a feedstock composition that is appropriate for producing biodiesel with good fuel qualities that are on par with ASTM standards^32,33^. An outstanding technique to produce green diesel is transesterification, which involves combining the catalyst with the oil and methanol^34,35^. Ester conversion is influenced by process variables such as temperature, molar ratio, catalyst amount, and retention time^36^. The catalytic efficiency of potassium hydroxide in biodiesel production yield was improved from 59.8 to 98.7%, subject to optimization^37^. Lowering production costs and increasing biodiesel output can be achieved through the optimisation of the transesterification process^38^. The literature confirms that the transesterification process has the potential to enhance biodiesel production as long as its parameters are optimised. Therefore, studying efficient experimental methodologies and computational machine learning techniques is essential to optimise and improve the efficiency of biodiesel production.

### Theory, potential utility and adaptability of EBOA, DTBO, and GWO approaches.

The concept of population-based search algorithms, namely EBOA and DTBO, has been conceived from human activities, while that of metaheuristic algorithms, most importantly GWO, originated from strategies of animals^39^.

EBOA is a population-based metaheuristic algorithm whose members are community individuals. The EBOA was developed to mimic the voting process to select the leader. The fundamental inspiration behind EBOA was the voting process, the selection of the leader, and the impact of public awareness level on the leader's selection. The EBOA population is guided by the search space under the leadership of the elected leader. EBOA's process is mathematically modeled in two phases: exploration and exploitation. The EBOA is a metaheuristic optimization algorithm inspired by the election process in democratic systems. It was proposed by Trojovský et al.^40^. EBOA simulates the election process where candidates (solutions) compete to become the leader (best solution). The fundamental inspiration of EBOA is the voting and election process in which people vote for their preferred candidate to elect the leader of the population. The EBOA steps in two phases: exploration, including the election process, and exploitation, including raising public awareness for better decision-making, are mathematically modelled.

The DTBO is a novel optimisation algorithm inspired by the process of driver training. The underlying concept behind the DTBO design is the process of learning to drive at a driving school and through driving coach training. DTBO is mathematically modeled in three phases: (i) training by the driving instructor, (ii) emulation of students from instructor skills, and (iii) practice. By incorporating this analogy into the optimization process, DTBO achieves a proper balance between exploration and exploitation and offers effective optimization solutions. This approach makes DTBO more proficient at exploring the search space and finding optimal solutions for various optimisation problems compared to other metaheuristic algorithms that rely solely on mathematical models. Moreover, the ability of DTBO to balance global and local search capabilities makes it a robust optimization algorithm with broad applicability. Therefore, DTBO surpasses other metaheuristic algorithms, such as PSO and JAYA, in significantly improving tracking time, reducing fluctuations, and achieving greater power output efficiency. Dehghani et al.^41^ proposed DTBO and reported that it mimics the process of adjusting driving parameters to optimize the performance of a vehicle, as seen in the economic dispatch problem^42^. The DTBO design was primarily influenced by how individuals learn to drive in driving schools and through instructor- training programs. The suggested DBOA has several advantages for challenging optimization problems, as well as its expected versatility in handling various types of optimization problems, given that many problems require more flexibility than DTBO can provide. Due to its mathematical foundation, DTBO can be utilised to address a variety of engineering optimisation problems, especially those with high dimensionality^43^.

The GWO algorithm is a new meta-heuristic optimization method inspired by the foraging social behavior of grey wolves. The GWO was first proposed by Mirjalili et al.^44^. The GWO algorithm mimics the leadership hierarchy and hunting mechanism of grey wolves in nature. Four types of grey wolves, namely alpha, beta, delta, and omega, are employed to simulate the leadership hierarchy.

EBOA, DTBO, and GWO models has been explored beyond biodiesel production optimization and into other areas of renewable energy or chemical process optimization. Table 1 highlights the studies related to the aforementioned algorithms. As observed, the potential applicability of the optimization algorithms of EBOA, DTBO, and GWO models have been explored individually or in combination in various engineering applications. Even though the limitations of adopting a single algorithm have been indicated, the hybridization of two or more algorithms has been noted to result in robust and reliable models in areas beyond biodiesel production optimization due to the broader impact and versatility of DTBO, EBOA, and GWO. However, numerous technical applications have examined binary and ternary models, as seen in Tables 2 and 3. As previously mentioned in Table 1, DTBO, EBOA, and GWO were used to model the engine characteristics of composite biodiesel/nanoparticle blends fuelled IC engines^8^; GWO was used to model the yield of Nahar oil methyl biodiesel^45^; ANN-GWO was explored in rice bran oil biodiesel^46^; GWO, IGOW, and MPR in estimating engine features of water-in-diesel emulsion-fuel powered IC engine^47^; RSM, ANN-GWO technique in approximating the yield of tobacco biodiesel^48^; GOA, WOA, ALO, and GWO in predicting fuel consumption and emission features of IC engines^49^; GP-GWO technique in viscosity prediction^50^; RSM-GWO  models explored in predicting engine and environmental features of canola oil biodiesel-EHN operated on a diesel engine^51^. GWO employs RSM based on BBD and CCD models derived empirical equations to optimize the biodiesel quality and yield of various feedstocks (refer to Table 1). GWO outperforms the RSM models in enhancing the biodiesel yield derived from canola oil^51^, abundant waste oil^52^, Nahar oil^45^, and animal waste fat-cottonseed-crude rice bran oils^8^. GWO's predicted transesterification conditions resulted in a higher biodiesel yield compared to the grasshopper optimization and firefly algorithms for niger seed oil^53^. The GWO-predicted values agreed adequately with the experimental datasets corresponding to performance and emission characteristics when fueled with biodiesel in diesel engines, compared to the Grasshopper Optimisation Algorithm and the Ant Lion Optimiser^49^. GWO's success in biodiesel research has led it to be an ideal choice for ethylic biodiesel derived from a ternary blend of neem oil, animal fat, and jatropha oil.

**Table 1:** Concise review of EBOA, DTBO and GWO on engineering applications and allied projects.

Engineering and allied applications	Analysis and optimization tools				Remarks	References
	EBOA	DTBO	GWO	Others		
Voting process for leader selection	$\surd$	$\times$	$\times$	$\times$	The suitability of EBOA showcased due to its enhanced global search capability	^40^
Interconnected power system incorporating electric vehicles	$\times$	$\surd$	$\times$	$\times$	Optimal regulator coefficient s and easy layout procedures are associated with DTBO -based controllers	^43^
Multi-objective problems	$\times$	$\surd$	$\times$	$\times$	The efficacy of DTBO has been proven due to its auspicious performance in convergence, diversity of obtained solutions, and computation time	^57^
PID controller	$\times$	$\surd$	$\surd$	$\times$	The simulation established that the DTBO-PID controller outperformed the GWO-PID controller	^58^
Power point tracking for a photovoltaic system	$\times$	$\surd$	$\times$	$\times$	Reductions in tracking speed and fluctuations in power output were observed from the employed DTBO	^59^
Power point tracking of wind turbine	$\times$	$\surd$	$\times$	Water Cycle Algorithm (WCA) and Particle Swarm Optimizer (PSO)	Better accuracy obtained from DTBO compared to WCA and PSO models	^60^
Solving Optimization Problems	$\times$	$\surd$	$\times$	$\times$	Better performance of DTBO established compared to ten competitor algorithms	^61^
Heat and power cogeneration system (HPCS)	$\times$	$\times$	$\surd$	$\times$	GWO established successively adopted in HPCS	^62^
Engineering Optimization (EO)	$\times$	$\times$	$\surd$	Elephant Herding Optimization (EHO) algorithm	GWOEHO outperformed the GWO	^63^
Maximum power point tracking (MPPT)	$\times$	$\times$	$\surd$	PSO	GWO-PSO established effective handling of partial shading in MPPT	^64^
Resolving global optimization problems	$\times$	$\times$	$\surd$	I-GWO and Ex-GWO	The outcome of the algorithms were successful for various problems	^65^

$$\times$$ = Absent, $$\surd$$= Present

**Table 2:** Overview of diverse Design of experiment like and MSSA in in low carbon fuel, automotive and biofuel industries.

Experimental technique	Protocols for green diesel production		Types of feedstocks	Variables	Response(s)	Optimisation methods	Significant remarks	Refs
	Methylic protocol	Ethylic protocol						
RSM	$\surd$	X	AWF-CSO-CRO mix	ME:HO = 3/1–15/1; C_d_ = 0.15–0.75 wt.%; R_te_ = 40–60 ^o^C; R_ti_ = 45–105 min	Yield	GWO	GWO outperformed RSM model in enhancing biodiesel quality and yield	^8^
BBD	$\surd$	X	Nahar oil (NO)	ME:NO = 7/1–13/1; C_d_ = 0.8–2.0 wt.%; R_te_ = 50–58 °C; R_ti_ = 100–200 min	Yield	GWO	Optimising NO biodiesel achieved through BBD and GWO models	^45^
BBD	$\surd$	X	Rice bran oil (RBO)	ME:RBO = 30–60; C_d_ = 0.5–1.0 wt.%; R_ti_ = 6–10 min	Yield	ANN-GWO	Reliability of the ANN-GWO model for optimising RBO biodiesel	^46^
X	NS	NS	X	W/D emulsion (0–30%);ES = 1000–3000 rpm	BP, BTE, BSFC, NOx	GWO, IGOW, MPR	Excellent exploratory capability of IGWO over GWO and MPR models established	^47^
RSM	$\surd$	X	Tobacco seed oil	ME:Tobaco seed oil = 4/1–8/1; C_d_ = 0.5–1.5 wt.%; R_ti_ = 40–80 min	Yield	ANN-GWO technique	GWO model for diversification of nocotiano based biodiesel prepared for tobacco industries	^48^
X	X	X	X	Fuel type and engine load	Thermal efficiency, fuel consumption, CO, HC, and NOx emissions	GOA, ALO, and GWO	GWO predicted values agreed adequately with the experimental datasets	^49^
x	$\surd$	X		ME:oil; C_d_; R_te_ ; R_ti_	Kinematic viscosity (KV)	Genetic Programming (GP)-GWO technique	Suitability of GP and GWO model proven for estimating KV of biodiesel	^50^
RSM	$\surd$	X	Abundant waste oil (AWO)	ME:AWOO = 4/1–8/1; C_d_ = 0.5–1.0 wt.%; R_ti_ = 40–80 min	Yield	GWO	GWO model outperformed RSM model in boosting AWO biodiesel from the eateries and food vendors	^52^
RSM	$\surd$	X	canola oil (CO)	EHN = 0–1000 mg/L; ES = 1600– 4400 rpm)	BP, BP, BTE, BSFC, NO_x_, CO_2_,	GWO	GWO slightly outperformed RSM model in predicting performance and emission emission of IC engine operated on CO biodiesel	^51^

ME:HO = methanol/hybrid oil molar ratio; C_d_ = catalyst dosage; R_te_ = Reaction temperature; R_ti_ = Reaction time; AWF-CSO-CRO = animal waste fat, cottonseed, and crude rice bran oils; BBD = Box Behnken design; *NS* = Not specified; *EHN* = 2-ethylhexyl nitrate; IGOW = intelligent grey wolf optimizer; MPR = multivariate polynomial regression; W/D = Water-in-diesel emulsion fuel.

**Table 3:** Overview of diverse human- based MSSA in engineering applications.

Engineering applications	Objective	RSM	Human- based metaheuristic soft computing	Remarks	Refs
Piezoelectric nonlinear system	Accurate characterization of the nonlinear piezoelectric hysteresis x systems,	X	DTBO algorithm	The DTBO algorithm outperformed Antlion Optimization algorithm	^54^
Diverse Hybrid Power System (DHPS)	Optimal controller coefficient in the controller’s steady operation and frequency regulation capability and prevention of its complex layout procedure	X	DTBO algorithm	The improvement of the controller of DHPS established	^55^
PID controller (PC)	Optimal tuning of PC in DC motor speed control	X	DTBO, Harris Hawks optimization, GWO, Atom Search optimization and Sine–Cosine Algorithm	DTBO outperformed understudied HBMSC	^45^
Engine performance and emission of hybrid biodiesel	Optimal conditions for engine features of IC	X	EBOA, DTBO and GWO	EBOA, DTBO outperformed GWO in IC operated on waste coconut and fish biodiesel	^56^
Photovoltaic system	To attain global maximum power point tracking (MPPT) with high convergence ability	X	DTBO and JAVA	Effectiveness of DTBO for better MPPT technique for PV systems established	^59^

X = Not Applied.

As highlighted in Table 3, some of these algorithms include the DTBO algorithm in the piezoelectric nonlinear system^54^; the DTBO algorithm in the diverse hybrid power system^55^; the EBOA, DTBO, and GWO models in the engine performance and emission of hybrid biodiesel^56^; and the DTBO and JAYA in the photovoltaic system^41^. GWO, DTBO and EBOA determined identical optimal parametric conditions for improved engine performance and emission characteristics for BOFs (waste coconut and fish oil)^56^. However, EBOA and DTBO require less computation time than GWO to determine the ideal optimal parametric conditions. The proven efficiencies (computationally efficient and determining global optimal condition) of DTBO, EBOA, and GWO algorithms in distinguished applications have led us to use them for achieving higher conversion of ethylic biodiesel yield from ternary feedstock oils.

Examining the full research studies reveals that the production of ethylic biodiesel from ternary generational feedstock oil (case study of NFJO) has not been investigated and predicted using RSM and three unique population-based stochastic search algorithms (DTBO, EBOA, and GWO). In addition to establishing a correlation between ethylic yield and ethanolysis operating parameters, this needs to be investigated in order to minimise computation time and effort.

### Gaps in knowelgde, novelty, motivation and objective of the study

The biodiesel and automobile sectors have used conventional, heuristic, and inefficient stochastic technologies to predict, model, and improve the production of green fuel by identifying the best solutions with minimal computing time and effort. Upon thorough examination of the literature, it can be observed that only methylic biodiesel derived from ternary oil has been studied^8^.The biodiesel stakeholders and experts would be tasked with anticipating, modelling, and scaling up the ethylic route to maximise production, as the methylic route has not demonstrated environmental benefits and failed to acknowledge the renewable nature and full biobased character of the ethylene route proposed for this study.The nonlinear relationship between the reaction parameters and responses has made it challenging to predict the influence of factors, even though biodiesel production requires experimentation. In an attempt to close knowledge gaps and expand the body of knowledge in science and engineering coupled with computer-based data analysis, this has led to the adoption of metaheuristic stochastic search algorithms (MSSA), such as GWO, DTBO, and EBOA, respectively. The lack of resilient, reliable, and consistent models has distorted the expected overall environmental benefits of low carbon production from ternary abundant oils.

The following tasks were undertaken to address the gap in relevant research within the existing literature and to improve the previously discussed ethyl yield: (i) central composite rotatable design of RSM was utilised to investigate the simultaneous influence effects of catalyst amount (0.65–1.15 wt.%), reaction temperature (55–65 °C), reaction time (45–75 min), and ethanol to oil molar ratio (5–7) on the yield of produced NFJOEE. (ii) The key and interaction effects among reaction variables impacting the ester conversion were analysed and the optimal conditions for alkaline ethanolysis were determined using the RSM approach. (iii) Optimal response variables described in terms of computation time and iteration by RSM, GWO, DTBO, and EBOA, convergence towards a global fitness. (iv) The NFJOEE fuel characteristics produced under optimal parameters were analyzed according to biodiesel standards. (v) Cost analysis of lab-scale NFJOEE production was determined for biodiesel. (vi) Develop correlations for the densities and viscosity of NFJOEE + Automotive gas oil/diesel fuel blends.

## Materials and methods

### Reagent, equipment, NFJO analysis and its ethylic production

For the purpose of producing ethylic biodiesel from NFJO with ethanol as the alcohol and KOH as the catalyst, jatropha, neem oils, and animal fat were obtained from a local slaughterhouse and an indigeous laboratory in Nigeria. The investigation employed high-purity analytical grade chemicals and reagents, as shown in Table 4, which were purchased from a local vendor in Edo State, Nigeria. Table 5 contains a list of all the primary equipment used in this study.

**Table 4:** Adopted major chemicals.

Substance	Formula	Molecular Weight g/mol	Specific Gravity	Maker	Purity%
Ethanol	C_2_H_5_OH	46.07	0.789	JHD, China	99.6
Potassium hydroxide	NaOH	40	2.13	JHD, China	99.9
Sulphuric acid	H_2_SO_4_	98	1.84	Loba Chemie, India	98
Benzene	C_6_H_6_	78.11	0.876	BDH, England	99.5
Phenolphthalein	C_20_H_14_O_4_	318.32	1.28	Kermel, England	50

**Table 5:** List of equipment.

Property	Equipment	Manufacturer
Viscosity	NDJ-5S Viscometer and	Anton Paar, UK
Density	SVM 3000 50 ml Pycnometer (Density Bottles)	Anton Paar, UK
Water bath	HJ-3D Constant Temperature Water bath	B. Brian, England
Magnetic stirrer	MS300 Constant temperature magnetic stirrer	Jinotech Sc. China
Drying	Vacutherm VT 6025 Vacuum Oven	Thermo Sc. NJ, USA

The ASTM standard was used to assess NFJO's basic properties such as density, viscosity, acidity, and saponification value, with Table 5 summarising the equipment and methods applied.

**Fig. 1:**
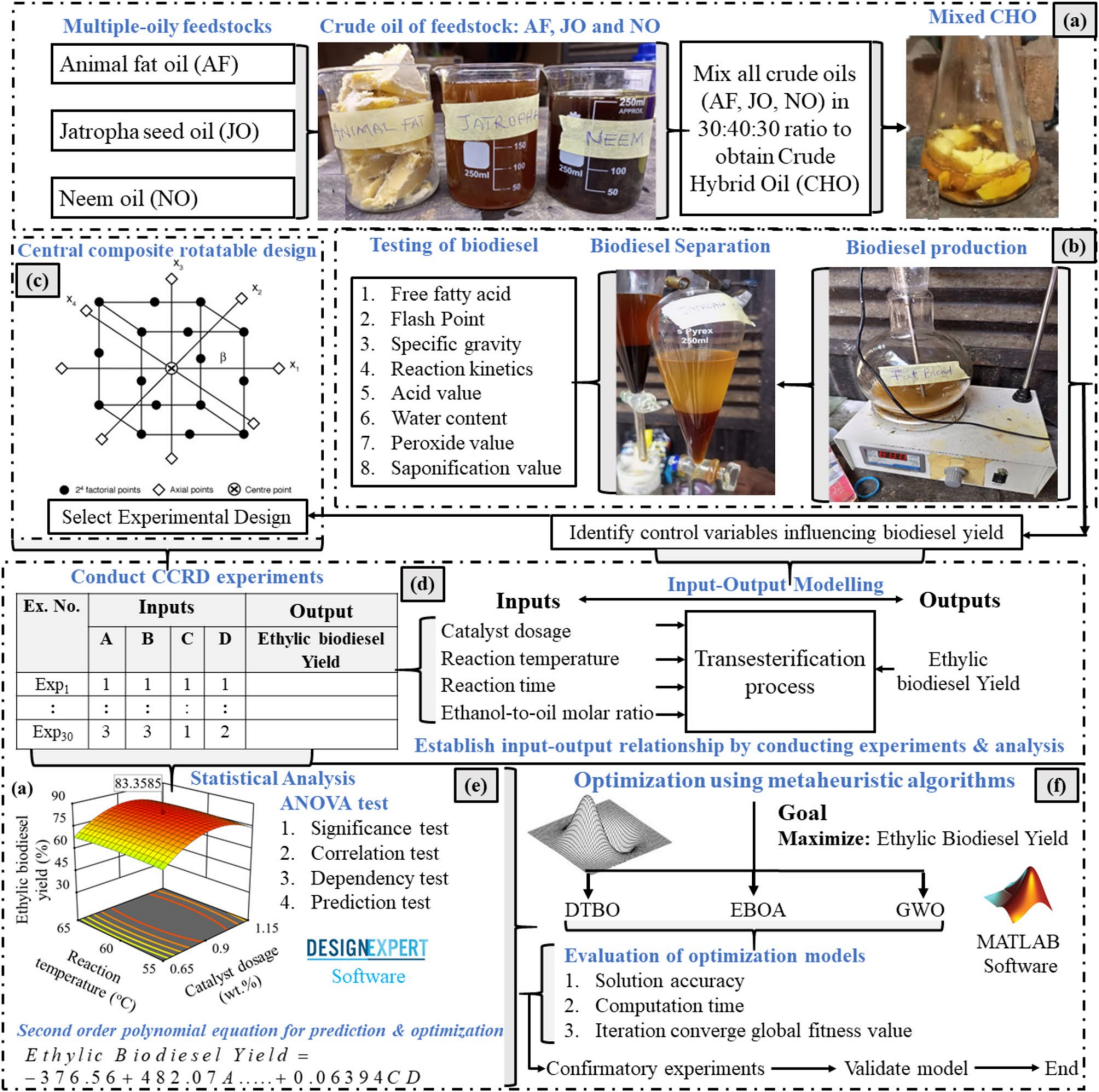
Schematic of the methodology in ethylic biodiesel from AF-NO-JO and its ternary robust modelling and optimization: (**a**) steps in preparation of mixed CHO from AF-NO-JO, (**b**) biodiesel production and testing fuel properties, (**c**) selection of experimental design, (**d**) experimental plan for input–output data collection, (**e**) statistical analysis of collected data, and (**f**) optimisation for maximized ethylic biodiesel yield using metaheuristic algorithms.

Figure 1 depicts the schematic of the methodology for pre- and post ethylic biodiesel production and analysis using RSM, GWO, DTBO, and EBOA technique. As oberved, the procedure entails: (i) Pre-treatment of high FFA NFJO and physicochemical properties, (ii) Ethylic biodiesel production from pre-treated NFJO via experimentation and DoE, (iii) Computational modelling approach with multiple inputs/responses, (iv) Analysis of AF-NO-JO ethylic biodiesel, (v) Fuel characterisation and GC–MS based analysis of NFJOEE at the optimal condition.

### Pre-treatment of high FFA NFJO and phyicochemical properties

**Fig. 2:**
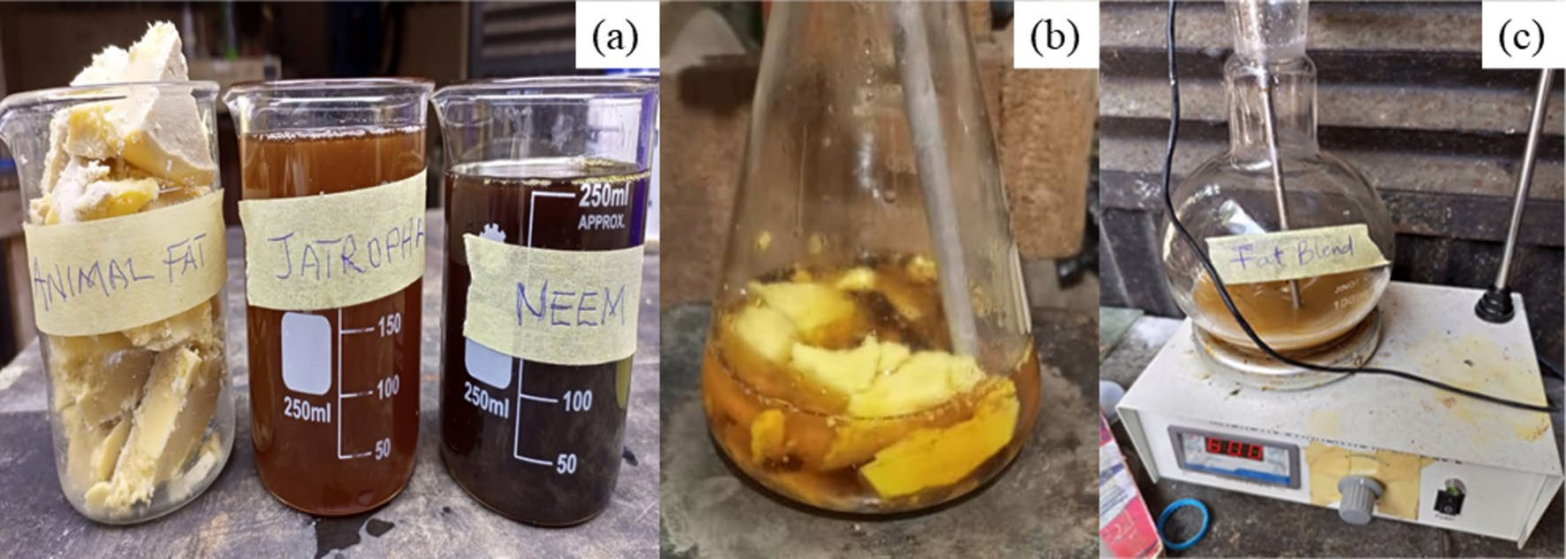
Pictorial view of NFJO: (**a**) AF, JO, NO; (**B**) high FFA NFJO blend; (**c**) esterification of NFJO.

Neem oil, animal fat, and Jatropha oil (NO, AF, and JO) were blended to produce NFJO in the precise proportion of 30:30:40, as previously described by^66,67^. 30 g of NO, 30 g of AF, and 40 g of JO were weighed into a 250 ml beaker and mixed with a magnetic stirrer (refer to Fig. 1a, b). After that, 40 g of recently extracted AF and a magnetic stirrer with a constant temperature setting of 70 °C were added to the mixture. Stirring was done to ensure a consistent homogeneous mixture of the novel ternary oil, which is NFJO. In a 1.0-L flask with a flat bottom, 500 g of NO, JO, and AF blend were weighed and combined with 25 g of methanol. Then, a catalyst of 1 wt.% of sulfuric acid (H_2_SO_4_) was added. The mixture was placed on a magnetic stirrer configured to constantly heat the mixture to 60 °C for an hour while agitating it at 1500 rpm. The %FFA was reduced to less than 1% by repeating the process as shown in Fig. 2 (a-c). The physicochemical properties of the NFJO were determined.

### Ethylic biodiesel of pre-treated NFJO via experiment and DoE

**Fig. 3:**
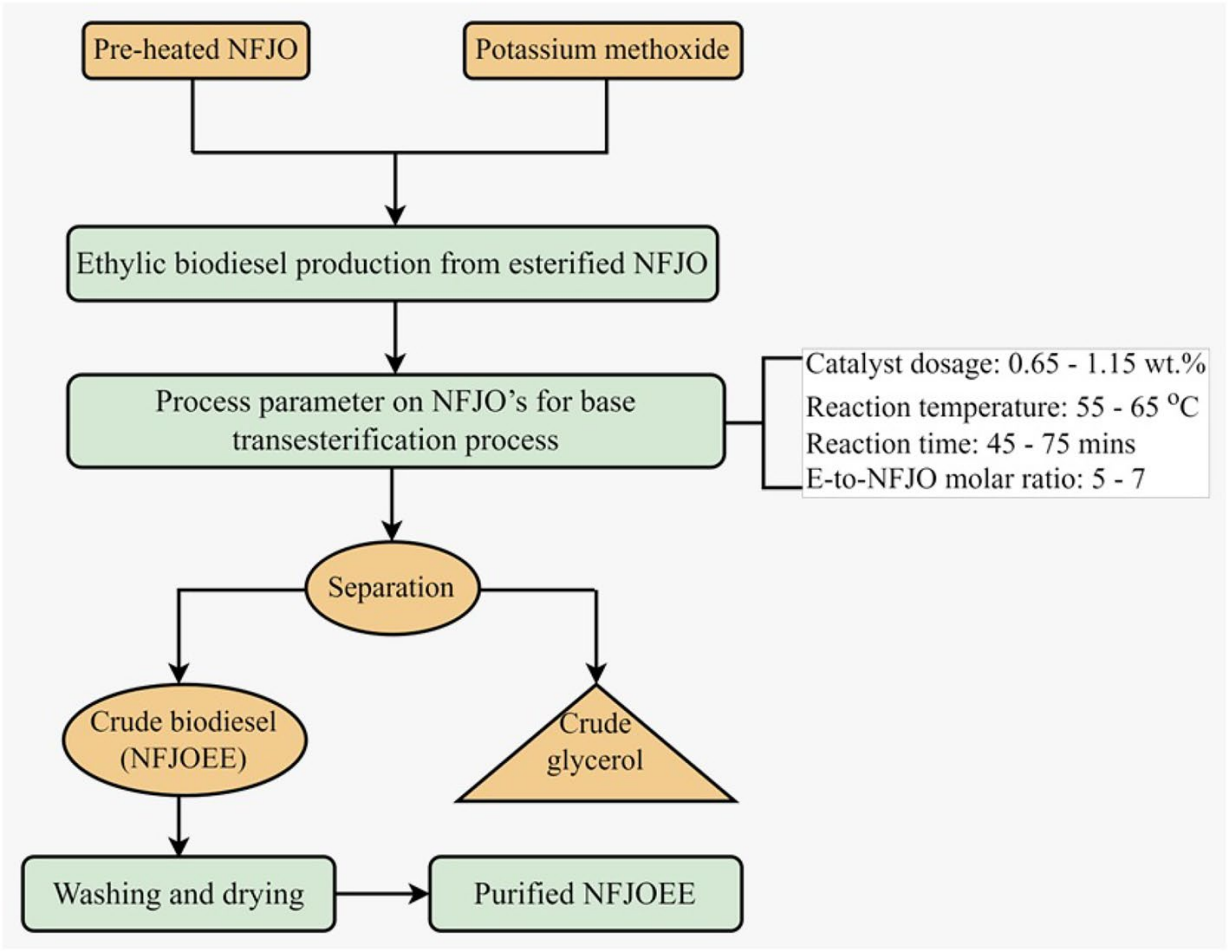
process specifications for producing ethylic biodiesel.

Central composite rotatable design (CCRD) was planned for experimentation to analyse the influencing variables such as reaction time, reaction temperature, catalyst dosage and ethanol-to-oil molar ratio on conversion of ethylic biodiesel (refer to Fig. 1c,d). Figure 3 depicts the process specifications for producing ethylic biodiesel from pre-treated NFJO. Pretreated NFJO underwent base ethanolysis, as previously described by^68,69^. In the presence of heat, mixing potassium hydroxide and ethanol resulted in the formation of a potassium ethoxide solution. Potassium ethoxide was added to hot esterified NFJO in a lab-scale reactor. The NFJOEE was allowed to settle after the transesterification operation was completed. Equation (1) was employed to determine the yield of NFJOEE for the respective runs.

1 $$Yield{\text{ of NFJOEE (\% ) = }}\frac{{M_{NFJOEE} }}{{M_{NFJO} }} \times 100 \,$$

### Theory of computational approach of models and multiple inputs/response

This section entails the theory with the mathematical context of empirical method and population-based MSSA including the RSM, DTBO, EBOA, and GWO techniques. However, three MSSA techniques namely GWO, DTBO, and EBOA were applied to determine the maximum ethylic biodiesel yield from a set of transesterification conditions. The use of a population of solutions, iterative search for optimal or nearly optimal solutions, and a balance between exploration (exploring new areas within the solution space) and exploitation (updating known good solutions) during the optimization task are common characteristics among the algorithms selected^56,70^. After its development in 2014, the GWO algorithm has been used to address a variety of issues^8,48,52,71^. However, since the DTBO and EBOA were developed in 2022, there is little evidence of their application in the literature for problem-solving^56^.

#### Modelling by RSM

The four factors influencing the yield of ethylic biodiesel were examined using transesterification studies. Four criteria led to the selection of the CCRD experimental plan for investigation and analysis. Table 6 presents the specifics of the factors and the operating levels for the experiments. 30 experiments (16 factorial, 8 axial, and 6 center) were analyzed, out of which 6 center point experiments were created for four factors by utilizing Eq. (2)^72^. The axis of each individual factor at a distance of ± α (α = 2 ^design variables/4^ = 2 for design variables = 4) serves as the basis for the axial point experiments. The independent variables were coded at five levels between − 2 and 2, and these coded levels and control variables were chosen for each component analysis^73^.

**Table 6:** Transesterification factors and levels.

Details	Transesterification variables (levels: low, medium, high)			
	Catalyst dosage (wt.%)	Reaction temperature (^o^C)	Reaction time (mins)	Ethanol to oil molar ratio
Notation	A	B	C	D
Ranges	0.65 to 1.15	55 to 65	45 to 75	5 to 7
+ α and − α	0.4 and 1.4	50 and 70	30 to 90	4 and 8

Input–output data collected from experiments were analyzed for parametric significance using ANOVA tests to derive empirical equation useful for prediction and optimization (refer to Fig. 1 d-e). Metaheuristic algorithms were applied to the derived regression equation to search for the maximum ethylic biodiesel yield subject to input variable constraints (refer to Fig. 1f).

2 $$\underbrace {N}_{Experiments} = \underbrace {2k}_{{Axial \, po{\text{int}} s}} + \underbrace {{2^{k} }}_{{Factorial \, po{\text{int}} s}} + \underbrace {{C_{p} }}_{{Centre{\text{ poins}}}}$$

#### Modelling by DTBO

Ni et al.^54^ described DTBO as a unique MSSA technique that emulates the driving training paradigm, involving learning and adaptation. The driving school is where the training paradigm begins, as a student driver chooses from a variety of instructors who subsequently offer advice and direction^55^. The trainee driver's goal is to become proficient in driving by using the instructor's method together with additional practice on their own. Investigators have a great chance to solve challenging cases with the help of the aforementioned framework. Three stages—exploration, exploitation, and optimisation—are represented mathematically in DTBO and are updated iteratively to produce optimal results^41^.

Phase 1: Training by driving instructor (Exploration)

This phase focuses on global search and exploration within the solution space. The DTBO update process involves learner drivers selecting the best-performing members as driving instructors from the DTBO population. Driving instructors guide other members (or learners) by imparting training and facilitating skill acquisition during the learning process. This approach ensures that population members explore distinct areas within the search space effectively resulting in better exploration capability and deriving global solutions^61^. The mathematical modelling of the first phase involves updating the member position according to Eq. (3).

3 $$\begin{gathered} \underbrace {{X_{{P_{1} ,i}} }}_{\begin{subarray}{l} \, New{\text{ position of i}}^{{{\text{th}}}} {\text{ candidate }} \\ {\text{ solution of Phase 1}} \end{subarray} } = \hfill \\ \left\{ {\begin{array}{*{20}c} {x_{{p_{1} ,i}} + rand[0,1] \, \bullet \, \left( {DI_{i} - rand \, I[1,2] \, \bullet \, x_{{p_{1} ,i}} } \right) \, \underbrace {{{\text{ OF}}_{DI,i} }}_{\begin{subarray}{l} \, Objective{\text{ function }} \\ {\text{value of driving matrix}} \end{subarray} } < \underbrace {{OF_{{p_{1} ,i}} }}_{\begin{subarray}{l} \, Objective{\text{ function value of }} \\ {\text{ previous position of Phase 1}} \end{subarray} }} \\ {\underbrace {{x_{{p_{1} ,i}} }}_{\begin{subarray}{l} \, previous{\text{ position of i}}^{{{\text{th}}}} \, \\ {\text{ candidate solution of Phase 1}} \end{subarray} } + rand[0,1] \bullet \left( {x_{{p_{1} ,i}} - \underbrace {{DI_{i} }}_{\begin{subarray}{l} {\text{ i}}^{{{\text{th}}}} \dim ens{\text{ion of }} \\ {\text{driver instruction matrix}} \end{subarray} }} \right) \, Else \, } \\ \end{array} } \right. \hfill \\ \end{gathered}$$

Phase 2: Modelling learner behaviour after driving instructor techniques (Exploration)

In the second phase, the learner driver imitates the skills, patterns, and driving techniques of the instructor. This process allows DTBO members to transition and shift to various positions in the search space, thereby enhancing the algorithm's exploration capabilities^41^. To mathematically simulate the said phenomenon, the updating of new positions is done using Eqs. (4–6).

4 $$\underbrace {{P_{t} }}_{\begin{subarray}{l} Patterning \, \\ \, Index \end{subarray} } = 0.01 + 0.9\left( {1 - \frac{{C_{I} (Current{\text{ iterations)}}}}{{M_{I} (Maximum{\text{ iterations)}}}}} \right)$$

5 $$\underbrace {{X_{{P_{2,i} }} }}_{\begin{subarray}{l} New{\text{ position of i}}^{{{\text{th}}}} {\text{ candidate }} \\ {\text{ solution of Phase 2}} \end{subarray} } = P_{t} \, \bullet \, \underbrace {{x_{{P_{2,i} }} }}_{\begin{subarray}{l} \Pr evious{\text{ position of i}}^{{{\text{th}}}} {\text{ candidate }} \\ {\text{ solution of Phase 2}} \end{subarray} } + \left( {1 - P_{t} } \right) \, \bullet {\text{ DI}}_{i} \,$$

6 $$X_{i} = \left\{ {\begin{array}{*{20}c} {X_{{P_{2,i} }} ,{\text{ OF}}_{DI,i} < \underbrace {{{\text{OF}}_{P2,i} }}_{\begin{subarray}{l} Objective{\text{ function value of }} \\ {\text{previous position of Phase 2}} \end{subarray} }} \\ {x_{{P_{2,i} }} ,{\text{ Else }}} \\ \end{array} } \right.$$

Phase 3: Personal practice (Exploitation)

In this phase, each learner driver aims to strengthen and refine their driving skills. This involves each driver learner focusing on personal practice to attain their personal best skill level, emphasising exploitation. They conduct a local search near their current position to determine the most advantageous location, showcasing the algorithm’s ability to find the best solutions. This phase includes generating a set of random positions close to each member, enhancing the solutions corresponding to the objective function value demonstrating their effectiveness in local search and exploitation^41^. Mathematically the positions are updated using Eq. (7).

7 $$\underbrace {{X_{{P_{3} ,i}} }}_{\begin{subarray}{l} New{\text{ position of i}}^{{{\text{th}}}} {\text{ candidate }} \\ {\text{ solution of Phase 3}} \end{subarray} } = x_{{P_{3} ,i}} + \left( {1 - 2 \, \bullet \, rand[0,1]} \right) \, \bullet { 0}{\text{.05 }} \bullet \, \left( {1 - \frac{{C_{i} }}{{M_{t} }}} \right) \, \bullet \, x_{{P_{3} ,i}}$$

#### Modelling by EBOA

EBOA is a novel MSSA technique that mimics the human electoral process. In the electoral process, community members select a leader through a voting phenomenon, where the elected leader impacts all members of society, including those who did not vote for them. The selection of the right candidates relies on the awareness level of community members. A more knowledgeable electorate (voters) tends to make better choices in candidate selection. In EBOA, the awareness of the electoral members or candidates increases the likelihood of selecting the most suitable leader. This concept of the election-voting process is mathematically modelled for solving complex problems, involving two phases (exploration and exploitation).

Phase 1: Voting process and holding elections (exploration)

Members of EBOA, drawing on their awareness and expertise in the electoral process, participate in voting for a candidate. This awareness is crucial for selecting quality leaders which are influenced by the value and quality of the objective function, a determinant in their choice. Individuals with more awareness contribute to improved objective function values (OFV)^40^. The mathematical representation of this process, including how it reflects the community's individual choices, is detailed in Eq. (8).

8 $$\underbrace {{AW_{i} }}_{\begin{subarray}{l} Awareness{\text{ of i}}^{{{\text{th}}}} \\ {\text{ candidate }} \end{subarray} } = \left\{ {\begin{array}{*{20}c} {\frac{{OFV \, _{{\text{i}}} - OFV \, _{Worst} }}{{OFV \, _{Best} - OFV \, _{Worst} }}} \\ { \, 1,{\text{ Else }}} \\ \end{array} } \right.{, }OFV \, _{Best} \ne OFV \, _{Worst} \,$$

The term OFV_i_ represents the objective function value of the i^th^ member. The OFV_best_ and OFV_worst_ represent the best and worst values of the problem domain. For a maximisation problem, the maximum value of OFV is considered the best and the minimum value of OFV is considered the worst, and vice versa.

In an election process, a minimum of two registered candidates (N_C_ ≥ 2) representing the top 10% of the most aware individuals in the community. These candidates are selected based on their individual awareness levels, with voters choosing the best candidate (C_1_) whose individual awareness level exceeds or is greater than of a random number. Conversely, less aware individuals are more likely to vote for other candidates. The mathematical formulation of the complete voting process (candidate selection and voting behaviour) is presented in Eq. (9).

9 $$\underbrace {{V_{i} }}_{\begin{subarray}{l} {\text{Vote of i}}^{{{\text{th}}}} \\ {\text{ person }} \end{subarray} } = \left\{ {\begin{array}{*{20}c} {C_{1} \, or \, best \, candidate{\text{ AW}}_{i} > rand_{i} } \\ {C_{k} \, or \, k^{th} \, candidate[2,3,....N_{C} ]} \\ \end{array} } \right.$$

After the EBOA voting process, the leader is selected based on the highest number of votes he/she received. This elected leader influences all community members, regardless of their vote, by guiding and inspiring the updating of their positions within EBOA. The crucial role played by the leader enhances the global search exploration capability by moving the population to distinguished search locations in the EBOA process. The process initiates with generating a random position for each member supervised by the leader. If new position determined improves the OFV, then the position is updated; otherwise, the previous best positions of members are retained for subsequent iterations. The update process in the EBOA is modelled using Eq. (10a,b).

10a $$\begin{gathered} \underbrace {{Y_{i,j}^{{new,Po_{1} }} }}_{\begin{subarray}{l} {\text{New position of i}}^{{{\text{th}}}} \\ {\text{ EBOA member }} \end{subarray} } = \hfill \\ \left\{ {\begin{array}{*{20}c} {Y_{i,j} {\text{ + rand[0,1] }} \bullet \, \left( {L_{j} - rand \, Integer[1{\text{ or 2}}] \times Y_{i,j} } \right)} & {\underbrace {{{\text{OFV}}_{L} }}_{\begin{subarray}{l} \, OFV{\text{ of a }} \\ {\text{ leader}} \end{subarray} } > \underbrace {{OFV_{i} }}_{\begin{subarray}{l} OFV{\text{ of a }} \\ {\text{i}}^{{{\text{th}}}} {\text{ member}} \end{subarray} }} \\ {Y_{i,j} {\text{ + rand[0,1] }} \bullet \, \left( {\underbrace {{Y_{i,j} }}_{\begin{subarray}{l} {\text{ i}}^{{{\text{th}}}} {\text{ candidate solution }} \\ {\text{ of j}}^{{{\text{th}}}} {\text{ dimension }} \end{subarray} } - \underbrace {{L_{j} }}_{\begin{subarray}{l} J^{th} {\text{ dimension }} \\ {\text{ of a leader}} \end{subarray} }} \right)} & {Else} \\ \end{array} } \right. \hfill \\ \end{gathered}$$

10b $$Y_{i} = \left\{ {\begin{array}{*{20}c} {Y_{i}^{new,Po1} {, }} \\ {Y_{i} ,} \\ \end{array} \begin{array}{*{20}c} { \, \underbrace {{{\text{OFV}}_{i}^{new,Po1} }}_{{OFV{\text{ of new position}}}} < \underbrace {{OFV_{i} }}_{{OFV{\text{ of a i}}^{{{\text{th}}}} {\text{member}}}}} \\ {Else} \\ \end{array} } \right.$$

Phase 2: Exploitation process by raising awareness among the public movement

In the election-voting process the awareness of society plays a vital role in making informed decisions. Individual thoughts activities and leaders contribute to increasing awareness. Mathematically the exploitation or local search produce a better solution in the EBOA process. Evaluating the objective function at a random position near each member in the search space accomplishes this task. If the new position gains a better OFV, update the members position. A better OFV signifies a successful local search and enhances individual awareness, aiding better decisions in subsequent iterations. This process of local search and its impact on awareness and decision-making is a leader-led initiative, where educating the public and raising their consciousness about various ideas and behaviors are key to determining better solutions to problems^40^. Mathematically the above task (raising public awareness) is represented using Eqs. (11, 12).

11 $$\underbrace {{Y_{i,j}^{{new,Po_{2} }} }}_{\begin{subarray}{l} {\text{New position of i}}^{{{\text{th}}}} \\ {\text{ EBOA member }} \end{subarray} } = Y_{i,j} { + }\left( {{1 - 2} \times {\text{rand[0,1]}}} \right) \, \bullet \, 0.02 \, \bullet \, \left( {1 - \frac{{Iteration{\text{ contour}}}}{{Maximum{\text{ iteration}}}}} \right) \bullet Y_{i,j}$$

12 $$Y_{i} = \left\{ {\begin{array}{*{20}c} {Y_{i}^{new,Po2} {, }} \\ {Y_{i} ,} \\ \end{array} \begin{array}{*{20}c} { \, \underbrace {{{\text{OFV}}_{i}^{new,Po2} }}_{{OFV{\text{ of new position}}}} < \underbrace {{OFV_{i} }}_{{OFV{\text{ of a i}}^{{{\text{th}}}} {\text{member}}}}} \\ {Else} \\ \end{array} } \right.$$

#### GWO modelling

GWO was developed to simulate the social hierarchy and hunting behaviour of grey wolves, encompassing both their prey search process and attacking strategy^44,74^. The algorithm is designed to determine the global solution for a problem by imitating the way wolves hunt in a pack (alpha α, beta β, delta δ, and omega ω)^75^. The hunting mechanism in GWO involves wolves encircling their prey guided by α: the leader of the pack represents the current best solution, β: follows the commands of α wolves representing the second-best solution, and δ: follows α and β wolves producing the third-best solution. The above concept is mathematically modelled mathematically to determine the best solutions as follows:

#### Encircling the prey

The α, β, and δ wolves positions act as a guide for ω wolves and update their position, and are mathematically represented using Eq. (13)^44,74^.

13 $$\underbrace {{\mathop Y\limits^{ \to } \left( {i + 1} \right)}}_{New \, position} = \, \underbrace {{\mathop Y\limits^{ \to }_{\alpha ,\beta ,\delta } {\text{(i)}}}}_{{Position{\text{ of }}\alpha , \, \beta ,{\text{ or }}\delta }}{ - }\underbrace {{\mathop {\text{Z }}\limits^{ \to } \bullet \mathop {\text{ C }}\limits^{ \to } }}_{Coefficient \, vectors}$$

##### Coefficient vectors

These vectors (Z and C) play a significant role in simulating the hunting behavior of grey wolves. The Z vector is used for diversifying the search space and controlling the exploration and exploitation phases in GWO. The computation uses a function that linearly decreases from 2 to 0 over successive iterations, facilitating a transition shift from wide-range exploration (searching solutions across wide area) to a more focused exploitation (fine-tuning search at promising areas) of the best solutions. The C vector offers random weight to the prey position (also referred to as the best-known position) that emphasises stochastic behaviour in the search process. This vector is typically computed with random values in each iteration, aiding in the random exploration of the search space and introducing unpredictability, mimicking the random hunting movements. Equations (14a) and (14b) provide the Z and C vectors, which are calculated and updated numerically to reflect the wolves' position during the search^44,74^.

14a $$\mathop Z\limits^{ \to } = { 2 } \cdot \underbrace {{\mathop {\text{a}}\limits^{ \to } }}_{\begin{subarray}{l} decreases{\text{ linearly}} \\ {\text{ from 2 to 0}} \end{subarray} } \cdot \, \mathop {rand_{1} }\limits^{ \to } [0, \, 1] - \mathop {\text{a}}\limits^{ \to }$$

14b $$\mathop C\limits^{ \to } = \, \left| {{2 } \cdot \mathop {rand_{2} }\limits^{ \to } [0, \, 1] \, \cdot \, \mathop X\limits^{ \to }_{\alpha , \, \beta , \, \delta } - \, \mathop {\text{X}}\limits^{ \to } (i)} \right|$$

##### Hunting, searching for prey, and convergence

The three best solutions obtained for α, β, and δ wolves were used to guide the optimal search for prey. Wolves randomly search for prey by adjusting their positions around the best solutions determined. The algorithm iteratively adjusts the positions of all wolves towards the best solutions. Over successive iterations the wolves converge to the optimal solution representing the prey^44,74^.

### Development of models for diesel blends and NFJOEE

Fuel blends were prepared with specific volume percentages of 10%, 20%, 30%, 40%, and 50%. The density of NFJOEE blends at 30 °C was measured using the Pycnometer (Anton Paar, UK) in accordance with ASTM D1298 test method. The kinematic viscosity was measured using a Viscosometer Batch (Anton Paar, UK) following ASTM D445. The average values for kinematic viscosity and density were reported. The densities and kinematic viscosities of the NFJOEE and diesel blends were correlated with the amount of biodiesel using the respective Eqs. (15a) and (15b).

15a $$D_{NFJOEE} = A_{S1} + B$$

15b $$KV_{NFJOEE} = A_{S1} + B$$

## Results and discussions

### Analysis of NFJO

Table 7 summarises the properties of NFJOEE. As observed, the acid value (18.573 mg KOH/g), saponification value (198.641 mg KOH/g), kinematic viscosity (34.13 mm^2^/s), FFA content (9.2865%), water content (0.13 wt.%), peroxide value (9.4 Meq/kg) and iodine value 108.4 Meq/kg are comparable with those in the literature^8,66^.

**Table 7:** Properties of AF-NO-JO and their mixture.

Properties	Units	TSO^66^	Ternary oil^8^	NFJOEE	Methods
Acid value	mgKOH/g	0.43	18.20	18.573	EN ISO 6618
Saponification value	mgKOH/g	134.42	182.42	198.641	EN ISO 3657
Viscosity	mm^2^/s	36.42	30.50	34.13	ASTM D445
FFA content	%	2.51	9.10	9.2865	Titration
Water content	wt.%	ND	0.02	0.13	
Peroxide value	M*eq*/kg	ND	2.83	9.4	
Iodine content	I_2_g/100 g	38	105.02	108.4	ASTM D5769

TSO = AF + CS + RB oil; Ternary oil: JC + HB + EGO.

### The compilation of data and determining its ideal conditions

The input–output data from the transesterification experiment, which were gathered using an RSM-based CCRD matrix, were examined to determine how individual factors and two-term factor interactions affected the yield of ethyl ester biodiesel produced. 3D surface and main effect plots, as well as ANOVA, were used in this analysis. The production of NFJOEE was found to be mathematically correlated with variables related to transesterification. The RSM model's coefficient of determination is assessed in order to potentially aid in the development of precise predictive models.

The experimental input–output data (ethylic transesterification variables-yield of NFJOEE) that correlate to the CCRD matrix are highlighted in Table 8. Design-Expert software was used to apply multiple regression analysis to the experimental input–output data that had been gathered. Equation (16), which displays the ethylic transesterification parameters (ETP) viz. catalyst dosage, ethanol to oil molar ratio, reaction temperature, and time versus NFJOEE, is a second-order polynomial expression that was developed.

16 $$\begin{gathered} Yield \, of \, NFJOEE \, \left( \% \right) \, \hfill \\ \, = - 376.56503 \, + \, 482.07897 \, A + 1.04738B + 4.74280C \, + 16.26966D - 174.07017A^{2} \hfill \\ \, - 0.044520B^{2} - 0.030612C^{2} - 4.26189D^{2} + 0.070750AB \, - \, 1.29788AC \, - 13.38675AD \hfill \\ \, + 0.003102BC \, + 0.694312BD + 0.063937CD \, \hfill \\ \end{gathered}$$

**Table 8:** CCRD matrix for the ethylic variables-yield of NFJOEE.

Exp. No	Catalyst dosage (wt.%)	Reaction temperature (^o^C)	Reaction time (mins)	Ethanol to oil molar ratio	Ethylic Biodiesel Yield (%)
1	0.9	60	60	4	63.629
2	1.15	55	75	7	56.739
3	0.9	60	60	6	75.425
4	0.65	65	75	7	75.422
5	1.15	65	75	5	71.008
6	0.9	50	60	6	80.316
7	0.4	60	60	6	31.402
8	0.9	60	90	6	74.225
9	0.65	65	45	7	48.944
10	0.9	60	60	6	84.536
11	0.65	65	75	5	63.541
12	0.65	55	45	7	40.712
13	0.65	55	75	7	64.449
14	0.9	60	60	6	84.536
15	0.65	65	45	5	44.146
16	0.9	60	60	6	84.536
17	1.15	55	75	5	70.564
18	0.9	60	60	6	84.536
19	1.15	65	75	7	61.107
20	0.65	55	45	5	50.135
21	1.15	55	45	7	49.093
22	0.9	60	60	8	66.667
23	0.9	60	30	6	35.064
24	0.9	60	60	6	84.536
25	1.15	55	45	5	73.135
26	1.15	65	45	5	64.831
27	1.4	60	60	6	45.954
28	0.65	55	75	5	65.082
29	1.15	65	45	7	65.675
30	0.9	70	60	6	75.171

#### Analysis of factors using plots and main effect plot

**Fig. 4:**
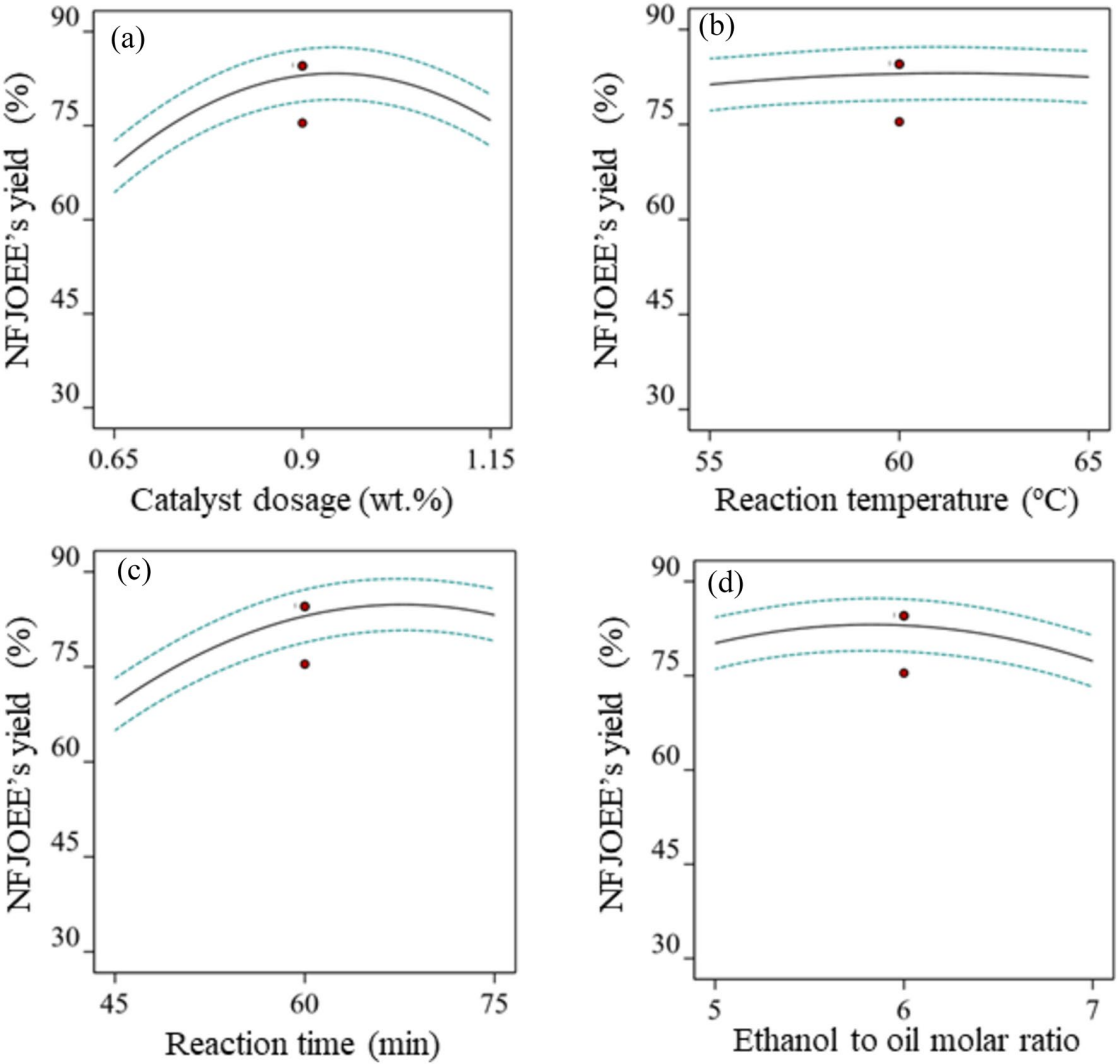
NFJOEE’s yield as a function of individual ethylic parameters: (**a**) NFJOEE’s yield vs. catalyst dosage, (**b**) NFJOEE’s yield vs. reaction temperature, (**c**) NFJOEE’s yield vs. reaction time, (d) NFJOEE’s yield vs. ethanol/oil molar ratio.

The main effect plots in Fig. 4(a-d) illustrate how each ETP and operating level affect the average yield values of NFJOEE. The non-linear impact of catalyst dosage on the yield of NFJOEE is seen in Fig. 4a. The catalyst enables and converts the triglycerides in oil and alcohol into biodiesel and glycerol, a byproduct, by lowering the activation energy required for the reactor and enhancing the reaction rate. Increased catalyst dosage increases NFJOEE's yield up to mid-values of 0.9 wt%, after which it declines. Because there are not enough active sites to fully adsorb reactants, a lower catalyst dosage (0.65 wt%) restricts the rate of reaction. The transesterification process between the reactant molecules (ethanol and triglyceride molecules) is enhanced by increasing the catalyst dosage (up to 0.9 wt%). It lowers the required activation energy for the transesterification reaction to complete its process, converting oil to yield^76^. The yield of the NFJOEE drops below the catalyst dosage's ideal concentration, which is reached when all reactant molecules are accommodated at active sites and the maximal reaction rate is achieved. This is explained by increased viscosity, the creation of a soap-like substance, the saponification of free fatty acids, and the challenge of separating the glycerol from the biodiesel^76,77^. The equilibrium phases reached, where the reactants have enough energy to actively interact with the catalyst and the reaction advances to the maximum rate, are what allow for the largest biodiesel yield, as shown in Fig. 4c^78^.

As can be observed in Fig. 4b, the yield of NFJOEE was determined to be relatively constant with a slight increase when the reaction temperature varied between their respective values. By giving reactant molecules more energy to collide and cross the activation energy barrier, reaction temperatures as high as 60 °C can quicken the transesterification or chemical reaction^77^. Jambingam et al.^78^ remarked that bubble formation tends to decrease the oil-ethanol interface and saponification formation occurs before complete transesterification. The NFJOEE yield showed a slight decrease at higher reaction temperatures, which was attributed to the vaporisation of ethanol from the reaction medium and reduced the proportion of ethanol to undergo transesterification reaction.

When the reaction reached the mid-values, the yield of NFJOEE increased, and as shown in Fig. 4c, the yield of biodiesel remained stable with a relatively small reduction at the end. A higher percentage of active catalysts are abundantly linked with the reactants as the transesterification reaction moves forward, resulting in the steady creation of biodiesel yield. This happens as a result of the oil's molecular structure requiring more time to perform a transesterification reaction in order to convert more biodiesel^77^. This oil contains higher energy saturated fatty acids. The reaction system reaches an equilibrium state, leading to catalyst deactivation^79,80^. The reasons for catalyst deactivation are as follows : (a) Saturated fatty acids have higher stability, reaching a quick equilibrium state where forward and reverse reactions are equal, leading to catalytic deactivation. (b) Saturated fatty acids might undergo side or reversible reactions that produce water or other compounds, which could deactivate the catalyst through hydrolysis. (c) Leaching occurs with an increase in catalyst dosage, resulting in a loss of catalytic activity and shifting the reaction towards equilibrium as the reaction rate slows.

The high stability of saturated fatty acids means the reaction can quickly reach equilibrium, where the rates of the forward and reverse reactions are equal, halting further conversion and causing apparent catalyst deactivation.

The NFJOEE's yield first increases with the ethanol-to-oil molar ratio, as seen in Fig. 4d, since additional ethanol accelerates the transesterification reaction. It should be noted that a higher ethanol to oil ratio facilitates the reaction, resulting in a higher biodiesel conversion by allowing the ethanol or reactant molecules to collide with the oil molecules.

The NFJOEE's yield drops below the midpoints of the ethanol to oil molar ratio for four reasons: (a) adding more ethanol does not change the already-achieved balance in favor of the conversion of ethanol into biodiesel (b) excess ethanol may prevent the separation of glycerol from biodiesel; (c) excess ethanol combined with a strong catalyst may cause saponification and soap formation, which may prevent the separation of biodiesel and glycerol; and (d) too much ethanol may make biodiesel more soluble in ethanol, causing it to stay in the ethanol phase rather than separate and reduce yield.

#### Surface plot analysis

**Fig. 5:**
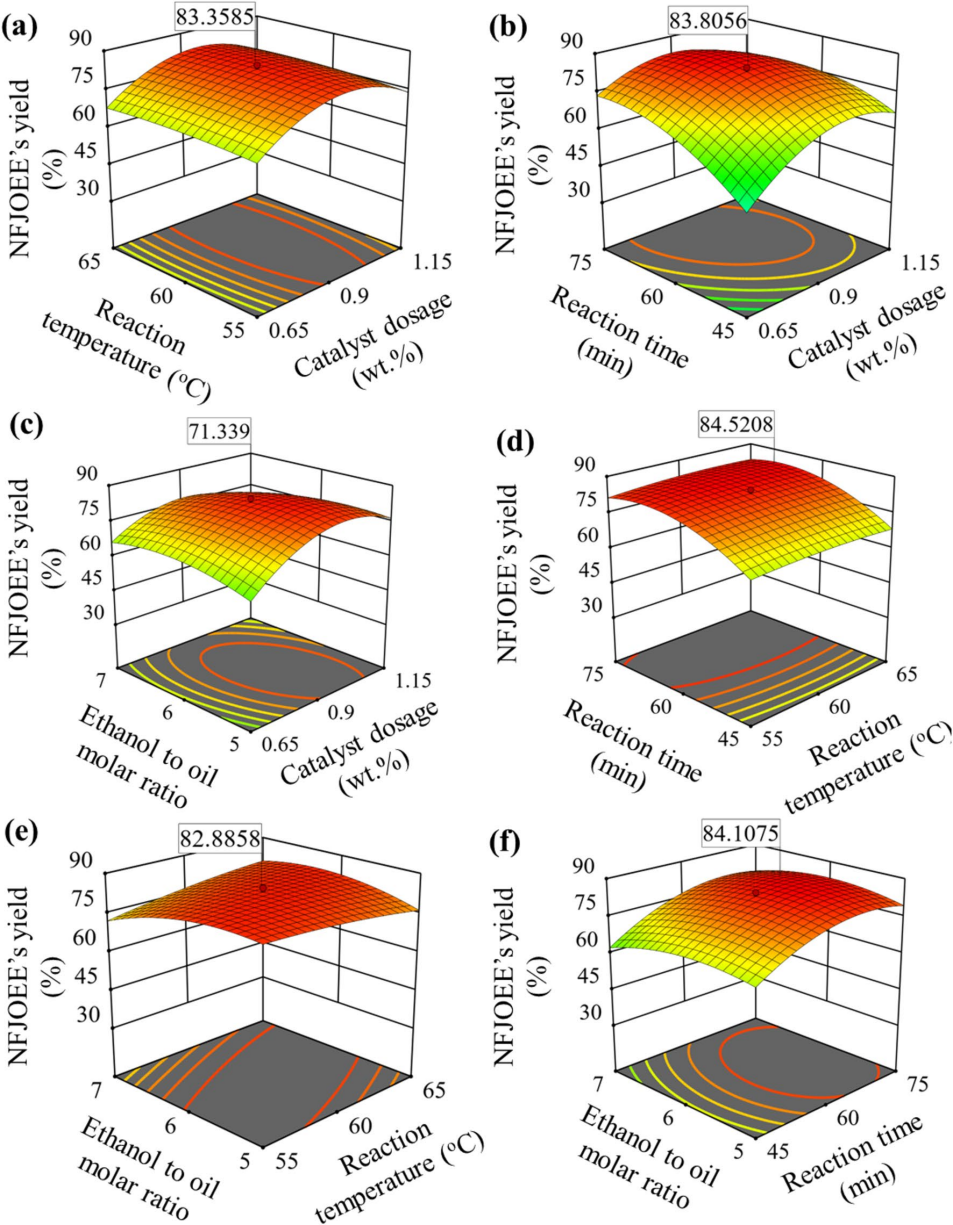
Surface plots of NFJOEE’s yield: (**a**) NFJOEE’s yield vs. catalyst dosage and reaction temperature, (**b**) NFJOEE’s yield vs. catalyst dosage and reaction time, (**c**) NFJOEE’s yield vs. catalyst dosage and ethanol-to-oil molar ratio, (**d**) NFJOEE’s yield vs. reaction temperature and reaction time, (**e**) NFJOEE’s yield vs. reaction temperature and ethanol-to-oil molar ratio, (**f**) NFJOEE’s yield vs. reaction time and ethanol-to-oil molar ratio.

Figure 5 presents 3D response surfaces for the yield of NFJOEE due to various ethylic variables. Figure 5a illustrates the interaction effects of catalyst dose and reaction temperature on ethylic biodiesel yield (after maintaining reaction duration and ethanol-to-oil molar ratio at mid-values equal to 60 min and (6). Near the mid-values of the catalyst dosage and reaction temperature, a maximum biodiesel yield of 83.36% was attained. Higher catalyst dosage values resulted in lower biodiesel yield; the reaction temperature led to the catalyst's accumulated bulk mass, saponification formation (emulsion and gel development), and solvent vaporization prior to the transesterification reaction's completion^76,77,81^.

As shown in Fig. 5b,a maximum biodiesel production of 83.81% was observed close to the mid-values of interaction terms, i.e., catalyst dosage and reaction time. The justification for the higher biodiesel yield up to the mid-points of catalyst dose and reaction time is that there is more available contact surface at the reactant mixtures with catalyst and more time allowed for the chemical reaction to occur^82,83^. The biodiesel yield decreased at higher catalyst loading and reaction time values because the reaction mixture became more viscous (i.e., the mono- and diglyceride molecules dissolved in the glycerol), making it harder to separate the biodiesel from the reactant mixture, initiate a reversible reaction, and deactivate the catalyst^84^.

The correlation between the ethanol-to-oil ratio and catalyst dosage and the yield of NFJOEE is shown in Fig. 5c. A biodiesel conversion of 71.34% was attained at or close to the middle values of the ethanol-to-oil molar ratio and catalyst dosage. By increasing the active contact surface at reactant mixes and allowing ethanol molecules to collide with oil, the catalysts speed up the ethylic process and enhance the yield of NFJOEE produced. Saponification and soap formation are caused by the solubility of ethanol in biodiesel, which makes it difficult to separate the biodiesel from the ethanol reaction phase mixture^85–87^.

As shown in Fig. 5d,a maximum NFJOEE yield of 84.52% was observed close to the mid-values of the interaction effects of reaction time and temperature. This might be justified by giving the transesterification reaction enough time to finish, which allows for the reaction mixture's diffusivity (from triglycerides to diglycerides and monoglycerides) to occur for the conversion of biodiesel with a high yield^88^. Longer exposure of the reaction mixture at higher temperatures causes the production of vapour phase, which lowers yield, as highlighted^89^.

The maximum NFJOEE yield of 82.89% is displayed in Fig. 5e, where the ethanol-to-oil molar ratio and reaction temperature are represented by interaction terms. The optimum higher NFJOEE yield values were noted in relation to each of their associated mid-values. The best possible circumstances were found for equilibrium phases, when the reaction temperature guarantees a faster chemical transesterification reaction (collision between reactant molecules between oil and ethanol) in the presence of ethanol^78,90^.

The maximum NFJOEE yield of 84.11% in Fig. 5f was found to be in proximity to the mid-values of the interaction effects of ethanol-to-oil molar ratio and reaction duration. For a higher conversion rate of biodiesel, ethanol's greater solubility in oil and solvent qualities guarantee that the ethanol completes the transesterification process^90^.

#### ANOVA for Quadratic model for NFJOEE’s yield

Analysis of variance is widely used for experimental data analysis providing detailed insights into process factors (linear: catalyst dosage, reaction temperature, reaction time, and ethanol-to-oil-molar ratio; square: catalyst dosage^2^, reaction temperature^2^, reaction time^2^ and ethanol-to-oil-molar ratio^2^; interaction: catalyst dosage x reaction temperature, catalyst dosage x reaction time, catalyst dosage x ethanol-to-oil-molar ratio, reaction temperature x reaction time, reaction temperature x ethanol-to-oil-molar ratio, reaction time x ethanol-to-oil-molar ratio) significance on outputs. The effect of factors (main, square, and interaction) on the ethylic biodiesel yield was statistically analysed for the preset 95% confidence level. The results of ANOVA for NFJOEE’s yield are presented in Table 9. The linear factors (such as catalyst dosage and reaction time) were found to have P-values less than 0.05, indicating a significant contribution towards ethylic biodiesel yield. P-values > 0.05 were recorded for reaction temperature and ethanol-to-oil-molar ratio, depicting a negligible contribution to ethylic biodiesel.

**Table 9:** Results of the analysis of variance for ethanol biodiesel.

Source	Sum of Squares	DF	Mean Square	F-value	p-value
Model	6563.80	14	468.84	20.32	< 0.0001
A: Catalyst dosage	328.75	1	328.75	14.25	0.0018
B: Reaction temperature	8.73	1	8.73	0.3784	0.5477
C: Reaction time	1197.98	1	1197.98	51.93	< 0.0001
D: Ethanol-to-oil molar ratio	48.81	1	48.81	2.12	0.1664
AB	0.1251	1	0.1251	0.0054	0.9423
AC	379.01	1	379.01	16.43	0.0010
AD	179.21	1	179.21	7.77	0.0138
BC	0.8663	1	0.8663	0.0375	0.8490
BD	192.83	1	192.83	8.36	0.0112
CD	14.72	1	14.72	0.6379	0.4369
A^2^	3246.47	1	3246.47	140.72	< 0.0001
B^2^	33.98	1	33.98	1.47	0.2437
C^2^	1301.25	1	1301.25	56.40	< 0.0001
D^2^	498.20	1	498.20	21.59	0.0003
Residual	346.07	15	23.07		
Lack of Fit	276.89	10	27.69	2.00	0.2297
Pure Error	69.18	5	13.84		
Cor Total	6909.87	29			

As illustrated in Fig. 5e, the major effect plot demonstrated a minor variation in NFJOEE's yield with the reaction temperature and ethanol-to-oil molar ratio. Higher F-statistic values for reaction time were recorded, depicting a major contribution towards ethylic biodiesel yield. The P-values of the square term of reaction time were found to be greater than 0.05, depicting a strong linear relationship with ethylic biodiesel yield. The interaction terms (catalyst dosage x reaction time, catalyst dosage x ethanol-to-oil-molar ratio, reaction temperature x ethanol-to-oil-molar ratio) were statistically significant. The resulting surface plots showed major variations in ethylic biodiesel yield (refer to Fig. 5 b, c, and e).Although the ethanol-to-oil molar ratio was found to be insignificant, the interaction with reaction temperature and catalyst dosage was statistically significant at a 95% confidence level. The effects of both the individual factors, i.e., catalyst dosage and reaction time, were significant, and their interaction effects on biodiesel yield were insignificant. Higher sum of squares and F-values were recorded for AC (catalyst dosage x reaction time) followed by BD (reaction temperature x ethanol-to-oil-molar ratio) and AD (catalyst dosage x ethanol-to-oil-molar ratio). The F-value of the model was found to be equal to 20.32, depicting its statistical significance. The model-determined coefficient of determination (R^2^) is 0.9499, depicting the model as statistically adequate. The model determined that the adjusted R^2^ (considering only significant terms: A, C, AC, AD, BD, A^2^, C^2^, D^2^) value was equal to 0.9032. Excluding insignificant terms from the model results in an imprecise input–output relationship and reduces prediction accuracy.

#### Optimised conditions for synthesised NFJOEE

Table 10 shows the optimum condition for the NFJOEE. As shown, a catalyst dose of 0.915%, a reaction temperature of 81.55 °C, a reaction time of 67.43 min, and a molar ratio of 5.99 between ethanol and NFJOE produced the maximum yield of TSOME (86.3%). With the modified experimental settings, the validation assessment resulted in an experimental yield of 86.4%. A 0.12% average error was identified. Since the error proportions in the forecast were consistent, the validation results indicated that the model was accurate.

**Table 10:** Justification test.

Catalyst dosage (wt.%)	Reaction temperature (^o^C)	Reaction time (min)	Ethanol-to-oil molar ratio	Predictedyield (%)	Experimental yield (%)	Error (%)
0.915	61.55	67.43	5.99	86.3	86.4	0.12

##### Comparison of the optimum conditions of NFJOEE with biodiesel literature

Table 11 lists the yield of NFJOEE under ideal circumstances. Differences in the fatty acid composition of the triglycerides in the oil, different reactor geometries, the type of catalyst, variations in the experimental conditions, and purification and washing during the biodiesel production process can all be considered as potential causes of the observed discrepancies in the yield of biodiesel.

**Table 11:** Optimum conditions of NFJOEE.

M(e)thyl esters of oil	Predictive tools	Optimum condition (OC)	Yield (%)	Remarks	Refs
AF-CS-RBO mix	RSM	M:O = 9:1,C = 0.45 wt.%, T_i_ = 105 min, T_e_ = 50 °C	94.2	Enhanced novel methyl biodiesel from AF-CS-RBO mix	^8^
WC-JC-PO mix	RSM	M:O = 9.86:1,C = 0.78 wt.%, T_i_ = 10.5 min, stirring speed = 478 rpm	96.81	Improved oxidation stability and cetane number obtained from methyl ester of WC-JC-PO mix	^91^
^1^JC-HB-EGO mix	RSM	M:O = 4.62:1,C = 4.16 wt.%, T_i_ = 69.7 min, T_e_ = 69.79 °C	93.5–96.9	Composite biodiesel was produced from JC-HB-EGO mix	^66^
HC-SI-BS-AY-NO mix	RSM	M:O = 4:1,C = 3 wt.%, T_i_ = 60 min, T_e_ = 59.40 °C	97.9	Enhanced methyl ester produced from HC-SI-BS-AY-NO mix	^92^
^2^LC-DS-LSO mix	RSM	M:O = 9:1,C = 90 wt.%, T_i_ = 90 min, T_e_ = 70 °C	96.63	Hybrid biodiesel was produced from LC-DS-LSO mix	^93^
*Arachis hypogaea*	Experimental design (ED)	E:O = 9:1,C = 90 wt.%, T_i_ = 90 min, T_e_ = 70 °C	95.49	Optimum yield (95.49) of Arachis Hypogaea ethyl ester produced at OC	^94^
Refined soybean oil (RSO)	ED	E:O = 3:1, C = 3 wt.%, T_i _= 15 min, water content = 20.3 wt.%	64.7	Optimal yield of RSO ethyl established	^95^
Animal fat waste (AFW)	A full 3^3^ factorial design	E:O = 7:1,C = 0.96 wt.% Ti = min, Te = 30 °C	83.5	Optimum yield of 83.5% was achieved for AFW ethyl ester using A full 3^3^ factorial design	^96^
NFJO	RSM	E:O = 5.99:1,C = 0.95 wt.%, T_i_ = 67.43 min, T_e_ = 61.56 °C	86.4	Complete enhanced ethyl ester bio-based composite biodiesel produced	Present study

AF-CS-RBO = animal waste fat, cottonseed, and crude rice bran (ACC) oils; 1JC-HB-EGO mix = Jatropha curcus, Hevea brasiliensis, and Elaeis guineensis oil; 2LC-DS-LSO mix = Luffa Cylindrical, Datura Stramonium, and Lagenaria Siceraria Oil Blend; WC-JC-PO mix = waste cooking oil – Jatropha curcas – Palm oil; HC-SI-BS-AY-NO mix = Hura creptian-Sesamum indicum-Blighia sapida-Ayo/Ncho oil.

#### Optimisation using population-based DTBO, EBOA & GWO algorithms

To maximize the yield of NFJOEE under transesterification conditions, three meta-heuristic algorithms were employed. Equation (16) describes the optimal search process under various constraints, derived from experimental data. The objective function for the GWO, DTBO, and EBOA algorithms was an empirical equation representing the production of NFJOEE based on transesterification variables.

All three algorithms were designed to find the optimal conditions for enhancing the ethylic biodiesel yield during the optimization process. The performance of the algorithms was compared based ton computation time and solution accuracy. The codes for the three algorithms (DTBO, EBOA, and GWO) were implemented using MATLAB software on a computer meeting the specified requirements (Intel Core i3 @ 1.2 GHz CPU and 4 GB RAM).

It is important to note that all three algorithms identified transesterification conditions (A: 0.915 wt.%, B: 61.55 oC, C: 67.43 min, D: 5.99) that maximize the NFJOEE yield at 84.983% (refer to Table 12). Experimental validation confirmed an 86.3% ethylic biodiesel yield under the ideal transesterification conditions.

**Table 12:** Computational test by optimisation algorithms.

Algorithms	Iteration converges to global fitness value	Computation time (population size and iterations: 50 and 1000)
DTBO	20	4 s
EBOA	7	4 s
GWO	985	34 s

**Fig. 6:**
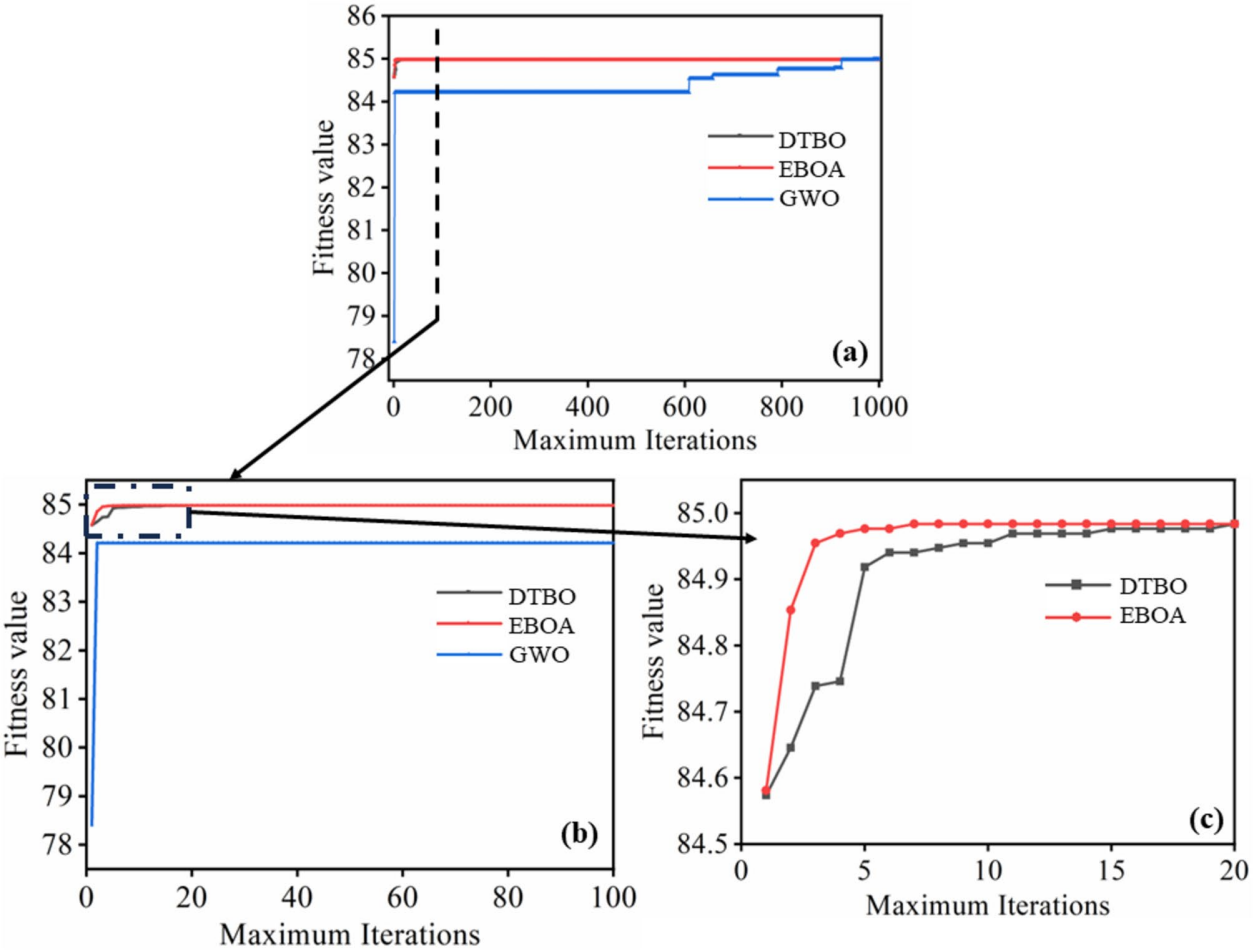
Fitness function values of three algorithms: (**a**) DTBO, EBOA and GWO for 1000 iterations, (**b**) DTBO, EBOA and GWO for 100 iterations, and (**c**) a) DTBO, and EBOA for 20 iterations.

By setting the population size and maximum number of iterations to 100 and 1000, respectively, the computational efficiency of the algorithms was evaluated.EBOA and DTBO outperformed GWO in terms of computation time for reaching the global fitness value (maximum NFJOEE). Although all three algorithms achieved a maximum fitness value of 84.983, the number of iterations required to converge to the global fitness value differed, with DTBO, EBOA, and GWO needing 20, 7, and 985 iterations, respectively (see Fig. 6a–c and Table 10). Additionally, DTBO and EBOA exhibited faster computation times of 4 s compared to 34 s for GWO. The superior performance of DTBO and EBOA over GWO can be attributed to factors such as the need for tuning algorithm -specific parameters in GWO, enhanced exploration capabilities in EBOA and DTBO, and a better balance between exploration and exploitation process

17 $$\begin{gathered} Yield \, of \, NFJOEE \, \left( \% \right) \, \hfill \\ \, = - 376.56503 \, + \, 482.07897 \, A + 1.04738B + 4.74280C \, + 16.26966D - 174.07017A^{2} \hfill \\ \, - 0.044520B^{2} - 0.030612C^{2} - 4.26189D^{2} + 0.070750AB \, - \, 1.29788AC \, - 13.38675AD \hfill \\ \, + 0.003102BC \, + 0.694312BD + 0.063937CD \, \hfill \\ \end{gathered}$$

Subjected to constraints are:

$$\begin{array}{*{20}c} {0.65 \le A:Catalyst{\text{ dosage}} \ge {1}{\text{.15}}} \\ { \, 55 \le B:{\text{Re}} action{\text{ temperature}} \ge {65}} \\ {45 \le C:{\text{Re}} action{\text{ time}} \ge {75}} \\ {5 \le D:Ethanol - to - oil{\text{ molar ratio}} \ge 7} \\ \end{array}$$

#### Fatty acid compositions and fuel assessment of NFJOEE obtained

Table 13 highlights the fatty acid ethyl ester composition of NFJOEE. As can be seen, aside from the greatest component of capric acid (25.87%), which is followed by oleic acid (21.07%), the remaining components include behenic acid (0.6%) and cerotic acid (0.12%). NFJOEE has a higher degree of unsaturation than saturation, which causes a longer premixed combustion and a higher peak pressure^97^.

**Table 13:** Fatty acid composition of NFJOEE.

Fatty Acid (Common Name)	Fatty Acid (IUPAC Name)	Concentration (%)
Capric acid	Decanoic acid, ethyl ester	25.87
Oleic acid	Octadecenoic acid, ethyl ester	21.07
Caprylic acid	Octanoic acid, ethyl ester	13.544
Palmitic acid	Hexadecanoic acid, ethyl ester	10.1
Lauric acid	Dodecanoic acid, ethyl ester	5.04
Arachidic acid	Eicosanoic acid, ethyl ester	2.46
Tridecylic acid	Tridecanoic acid, ethyl ester	0.92
Lignoceric acid	Tetracosanoic acid, ethyl ester	0.91
Behenic acid	Docosanoic acid, methyl ester	0.60
Pentadecylic acid	Pentadecanoic acid, methyl ester	0.31
-	Oxiraneoctanoic acid, 3-octyl-, methyl ester	0.31
Tricosylic acid	Tricosanoic acid, methyl ester	0.28
Undecylic acid	Undecanoic acid, methyl ester	0.24
Pelargonic acid	Nonanoic acid, methyl ester	0.21
Margaric acid	Heptadecanoic acid, methyl ester	0.13
Pentacosylic acid	Pentacosanoic acid, methyl ester	0.13
Cerotic acid	Hexacosanoic acid, methyl ester	0.12
	Total Conversion	82.244

The economic viability of synthesized biodiesel must meet global green diesel requirements to be assessed in that way. Certain requirements must be met to ensure the diesel engine's efficacy^52,98^. The characteristics of NFJOEE and other biodiesels are highlighted in Table 14. As can be seen, the kinematic viscosity of NFJOEE (5.72 mm^2^/s) was marginally higher than that of^8^ (4.33 mm^2^/s) and^99^ (4.48 mm^2^/s), but it was still marginally higher than that of diesel (4.48 mm^2^/s). It also exceeded EN 41,214's (3.5–5.0 mm^2^/s) specifications. When IC is powered by NFJOEE, there is no significant change because of the slight difference between NFJOEE's KV and those reported therein.

**Table 14:** Fuel properties of NFJOEE.

Properties	NFJOEE	Ternary oil methyl ester^8^^b^	HOEE^99^^c^	ASTM D6752	EN 14,214	Diesel fuel (B0)
KV @ 40 °C(mm^2^/s)	5.72	4.33	4.48	1.9–6.0	3.5–5.0	4.48
Density @ 30 °C (kg/m^3^)	886	880	862.9	NS	860- 900	861.8
Acid value (mgKOH/g)	0.35	0.12	ND	0.5 max	0.5 max	NA
Flash point (^o^C)	153	157	NR	130 min	120 min	76
Cloud point (^o^C)	− 3	− 8	NR	− 3 to − 12	< 0	− 9
Pour point (^o^C)	0	− 15	NR	< 0	< 0	− 15
HHV (MJ/kg)	41.1	NR	40.88	–	–	43.20

a, b, ternary oil methyl biodiesel; c.

*NA* Not Available, *NS* not specified.

The density of NFJOEE (866 kg/m^3^) was slightly higher than B0 (861.8 kg/m3) but in agreement with that of^8^ (880 kg/m^3^) and^99^ (862.9 kg/m^3^), as well as the EN 41,214 (850–900 kg/m^3^) standard. When injected, the fuel should not have a substantial impact on specific fuel consumption or fuel penetration, as indicated by the slightly higher density of NFJOEE compared to B0^100^. Although NFJOEE's acid value (AV) of 0.35 mg KOH/g was higher than^8^ 0.12 mg KOH/g, it nevertheless met ASTM D6751 and EN 14,214's (0.50 mg KOH/g) requirements. Because of the fuel's low acid value, NFJOEE cannot proceed through polymerization^101^.

**Fig. 7:**
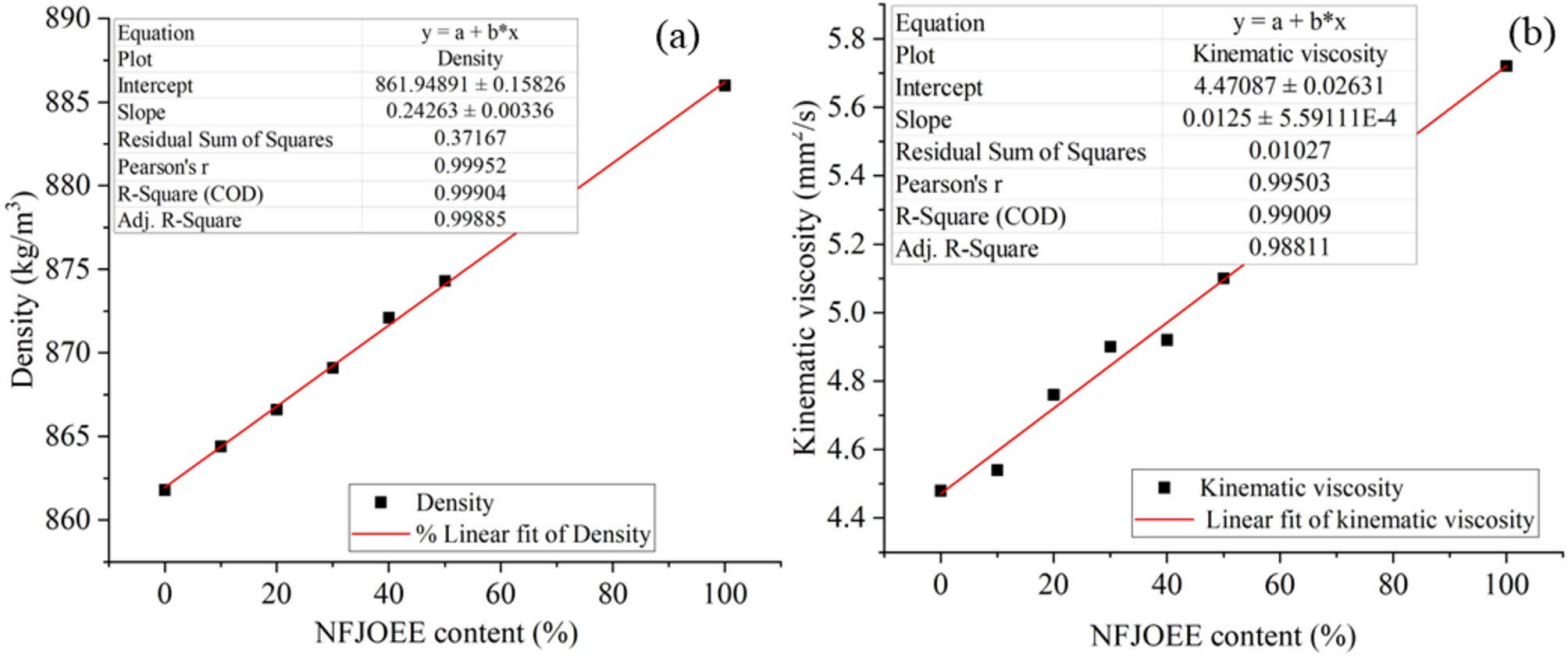
Density and viscosity models for NFJOEE: (**a**) Variation of density with NFJOEE content and (**b**) Variation of kinematic viscosity with NFJOEE content.

The changes in kinematic viscosity and density of the NFJOEE-diesel blends are shown in Fig. 7(a-b). For internal combustion engines, density is a very important quantity. High-density biodiesel can offset its lower heating value^102^. Due to this correction, biodiesel and diesel fuel can operate engines with similar performance characteristics^103^. As shown in Fig. 7a, the high R^2^ of 0.999 indicates that the linear equation ($0.24263x+861.95$) is detected as acceptable for forecasting density of NFJOEE-diesel blends as a function of biodiesel concentration. Bukkarapu and Baroutian et al. established similar correlations in their study^104,105^. Viscosity has been shown to impact injector pump atomization and flow^106^. Given the high R^2^ of 0.990, it is determined that the linear equation ($0.0125x+4.47087$) is suitable for modelling the KV of NFJOEE-diesel as a function of biodiesel content, as shown in Fig. 7b. Bukkarapu^105^. demonstrated that the one-dimensional model is appropriate for forecasting the KV of blends of NFJOEE and diesel.

Though NFJOEE's acid value (AV) of 0.53 mg KOH/g is slightly higher than Ganesha et al.‘^8^ value of 0.12 mg KOH/g, it is in line with ASTM D6752 and EN 14,214 specifications (0.5 max). The AV's adherence to the standards suggests that NFJOEE won't tend toward polymerization than Ganesha et al.'s^8^

The flash point (FLP) of NFJOEE (153 °C) was higher than B0's (76 °C) and in line with Ganesha et al.’s^8^ (157 °C), but it nevertheless satisfied both international standards' safety requirements. Biodiesel with a high FLP is less predisposed to fire vulnerability compared to diesel fuel^107^.

NFJOEE indicated pour point (PP) and cloud point (CP) values of 0 °C and -3 °C, respectively, which are higher than B0's values of − 9 °C and − 15 °C. These high PP and CP values are attributed to the saturated fatty esters' abundance in biodiesel, which may limit its wider use in cold climates^30^.

NFJOEE's heating values (HV) were marginally lower at 41.10 MJ/kg than B0's (43.20 MJ/kg). The fuel's greater oxygenated molecule is a contributing factor to the modest fall in HV value.

### Cost analysis

**Fig. 8:**
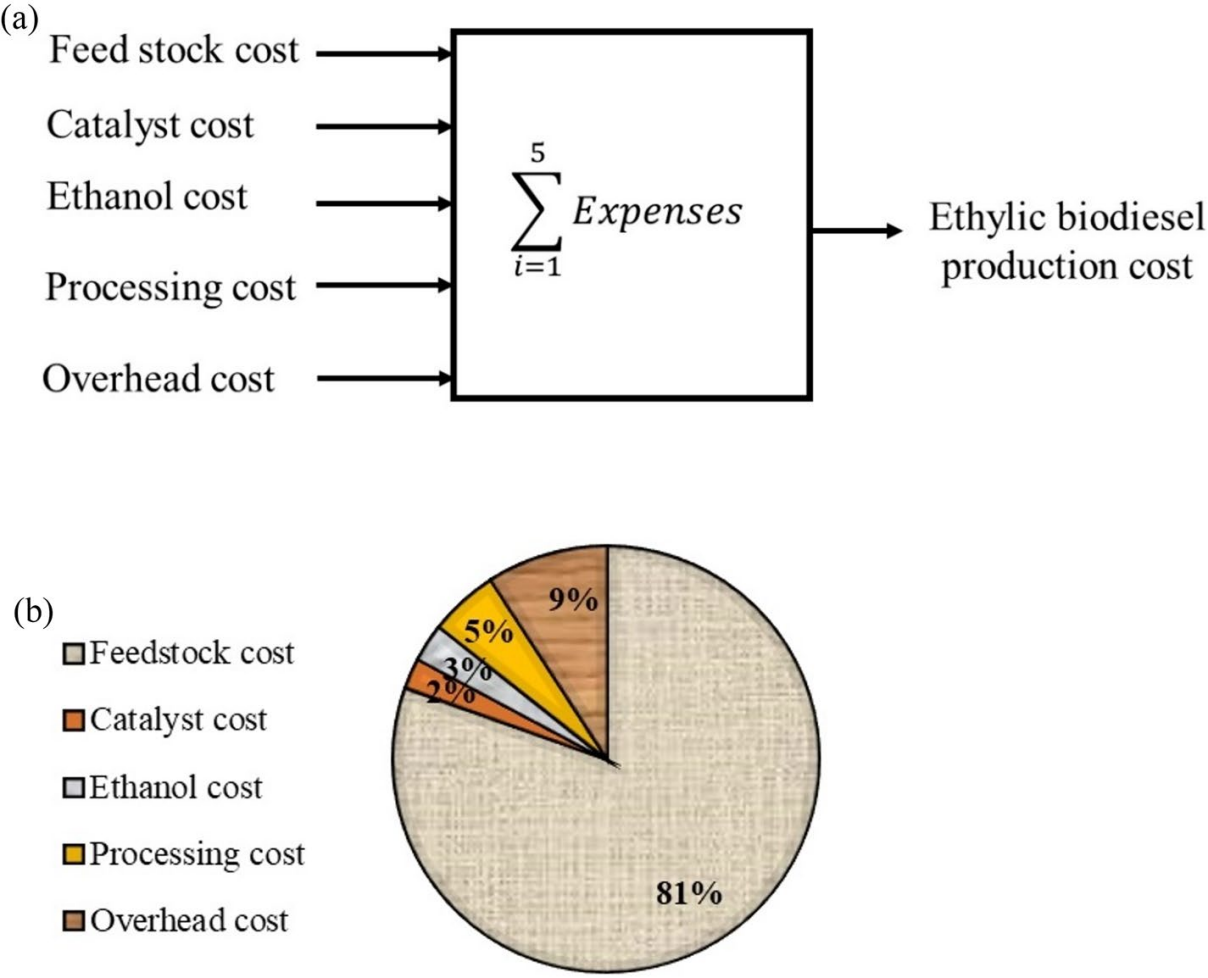
(**a**) Schematic for the cost estimation for the NFJOEE production, Fig. 8 (**b**) Cost assessment for NFJOEE production (Naira).

The methods proposed by researchers^108^ are used to assess the costs associated with biodiesel conversion from NFJO. The expenses associated with biodiesel production cost from a liter of NFJO include ethanol, KOH, power, process time and overheads (labour, equipment depreciation, maintenance and repair, insurance, and administrative expenses). Figure 8a illustrates a schematic for the mathematical computation of the biodiesel production cost from NFJO, while Fig. 8b shows the cost comparison of the biodiesel production cost component from NFJO.

The NFJOEE production cost associated with the present study help industries assess their practical utility considering all essential details presented in Table 15. The calculated overall production cost per kg of biodiesel is $0.9328. Figure 8a,b show the biodiesel production costs that indicates the cost of feedstock is the primary expense, accounting for 81% of total costs. Costs associated with catalysts and ethanol are secondary, while processing and overhead represent the smallest shares. The cost of feedstock is the most significant factor in biodiesel production, suggesting that securing affordable and consistent feedstock supplies is crucial for economic viability.

**Table 15:** Computation of biodiesel production cost.

Step	Description	Amount (in USD)
Feedstock	Oil cost for 1 kg of biodiesel production = (quantity × neem oil cost per kg + quantity × animal fat cost per kg + quantity × Jatropha oil cost per kg) = (0.3 * $ 0.84 + 0.3 *$ 0.6 + 0.4 * $ 0.78)	$ 0.75
Catalyst dosage consumed for 1 kg of biodiesel	0.00915 kg	$ 0.022
Ethanol required for 1 kg of biodiesel	0.02953 kg	$ 0.027
Processing cost	Transesterification time (h) × units consumed × cost per unit = (1.124 h × 0.8 × $ 0.054)	$ 0.049
Net cost (Feedstock + Catalyst + Ethanol + processing)	($ 0.75 + $ 0.022 + $ 0.027 + $ 0.049)	$ 0.848
Overhead cost (10% of net cost)	10% of $ 0.848	$ 0.0848
Biodiesel production cost per kg	Cost (net + overhead) = 0.848 + 0.0848	$ 0.9328

### Cost of NFJOEE production

Table 16 depicts the cost of biodiesel production using different feedstocks. The estimated production cost of NFJOEE ($0.9328 per liter) is lower when compared with the prices reported in the literature^109–115^. The NFJOEE processing costs are comparable to, yet lower than, those of conventional diesel fuel. Furthermore, Fig. 8b depicts the feedstocks alone account for 81% of the overall production cost. Reducing feedstock costs can be a strategic focus for cost management and operational efficiency^116^. Significant cost reduction potential exists in lowering feedstock costs through better price negotiations or finding cheaper alternatives^117^. This could lead to a competitive price advantage in the market. The calculated overall production cost of NFJOEE biodiesel is $0.9328 per kg, which could be further reduced by scaling up production and commercialization.

**Table 16:** Cost comparison of NFJOEE and other feedstock-derived biodiesels.

Feedstocks	Biodiesel cost (USD)	Refs
Municipal solid waste	1.47/liter	^109^
WCO	0.23–0.66/liter	^110^
	0.8/liter	^114^
Sludge	0.67–1.07/kg	^111^
HSO and TSO	1.26/liter	^98^
Diesel	1.06/liter	Price @ February 2024
Karanja oil	1.23 /kg	^112^
Castor	0.28/liter	^113^
*Spirulina platensis* oil	1.16/kg	^115^
NO + AF + JO	0.9328/kg	Present study

## Conclusion

In this study, sustainable resource management using environmentally friendly ethanol and ethylic biodiesel from ternary (neem, animal fat, and jatropha) oil (NFJO) mixed with a 30:30:40 volume proportion was explored on a lab scale with the help of the Central Composite Rotatable Design (Influence of ethylic variables such as ethanol-oil-molar ratio, catalyst dosage, reaction temperature, and time on the yield NFJO ethyl ester/ NFJOEE) coupled with cutting-edge population-based algorithms (PBAs) like DTBO and EBOA with GWO. The cost of NFJOEE was estimated. Models were developed to determine the densities and viscosities of NFJOEE-diesel fuel blends. The following can be deduced from this study in order to obtain a robust study in the near future: (i) technological and logistical approach for scaling up the process from a laboratory to an industrial scale; (ii) performance, emission, combustion, and exergetic indices of NFJOEE-butanol doped with nanoparticles; and (iii) varied ratios of neem oil, animal fat, and jatropha oil for ensuring availability, enhancing biodiesel yield, and quality should be further investigated. The objective conclusions drawn from the present work are:

- The yield of NFJOEE is not significantly affected by fluctuations within its operational levels, as indicated by the insignificance of the ethanol-to-NFJO molar ratio and reaction temperature. The yield of NFJOEE increased linearly with the variation in response time.
- The CCRD model exhibits a better coefficient of determination equal to 0.9499, indicating the model will be useful if employed for prediction and optimization. The insignificant terms (ethanol-to-oil-molar ratio, reaction temperature, reaction temperature^2^, catalyst dosage x reaction temperature, reaction time x reaction temperature, reaction time x ethanol-to-oil-molar ratio) need not be removed from empirical equations, which not only reduce prediction accuracy but also result in an imprecise input–output relationship.
- Three meta-heuristic population-based algorithms that use common features (iteratively searching for optimal solutions and balance exploration and exploitation during the search process) were applied to solve the optimization problem. EBOA, DTBO and GWO algorithms locate identical transesterification conditions (catalyst dosage: 0.915 wt.%, reaction temperature: 61.55 °C, reaction time: 67.43 min, ethanol-to-oil molar ratio: 5.99) that could maximize ethylic biodiesel yield analytically to 84.98%. The confirmation experiments yielded 86.3% of ethylic biodiesel yield corresponding to optimal transesterification conditions. Computationally, EBOA (4 s and 7 iterations) outperforms DTBO (5 s and 20 iterations) and GWO (985 iterations and 34 s) in converging solutions to locate global fitness values. GWO requires tuning of algorithm-specific parameters, unlike DTBO and EBOA. Furthermore, during optimal search, DTBO and EBOA showed better balance with exploration and exploitation. The results can be directly deployed for large-scale biodiesel production in industries.
- The NFJOEE fuel's characteristics agreed with the ranges of the EN 14,214 and ASTMD6751 requirements. It was determined that NFJOEE has a commercial value of (0.9328 USD/l).
- The density and kinematic viscosity models of the NFJOEE-diesel blends were found to be well-suited to the linear connection with high degree coefficient of determinations.

## Data Availability

The data is available within the manuscript.
